# The *Francisella tularensis* LVS Δ*pdpC* mutant exhibits a unique phenotype during intracellular infection

**DOI:** 10.1186/1471-2180-13-20

**Published:** 2013-01-29

**Authors:** Marie Lindgren, Jeanette E Bröms, Lena Meyer, Igor Golovliov, Anders Sjöstedt

**Affiliations:** 1 Department of Clinical Microbiology, Clinical Bacteriology and Laboratory for Molecular Infection Medicine Sweden (MIMS), Umeå University, Umeå SE-901 85, Sweden

**Keywords:** *Francisella tularensis*, Type VI secretion, Cytopathogenicity, Intracellular replication, PdpC

## Abstract

**Background:**

A prerequisite for the virulence of the facultative intracellular bacterium *Francisella tularensis* is effective intramacrophage proliferation, which is preceded by phagosomal escape into the cytosol, and ultimately leads to host cell death. Many components essential for the intracellular life cycle are encoded by a gene cluster, the *Francisella* pathogenicity island (FPI), constituting a type VI secretion system.

**Results:**

We characterized the FPI mutant Δ*pdpC* of the live vaccine strain (LVS) of *F*. *tularensis* and found that it exhibited lack of intracellular replication, incomplete phagosomal escape, and marked attenuation in the mouse model, however, unlike a phagosomally contained FPI mutant, it triggered secretion of IL-1β, albeit lower than LVS, and markedly induced LDH release.

**Conclusions:**

The phenotype of the Δ*pdpC* mutant appears to be unique compared to previously described *F*. *tularensis* FPI mutants.

## Background

Gram-negative bacteria utilize a variety of secretion systems to colonize and invade eukaryotic hosts. The most ubiquitous of these is the recently described type VI secretion system (T6SS), which appears to exist as a cluster of 15-20 genes that are present in more than 25% of all bacterial genomes [1,2]. The T6SS is a sophisticated protein export machine of Gram-negative bacteria capable of targeting effector proteins into host cells in a cell to cell contact-dependent manner, but also with the unique propensity to confer lytic effects on other bacteria [3-6]. Some of the T6SS components are evolutionarily related to components of bacteriophage tails and it was recently demonstrated that active protein secretion by *Vibrio cholerae* requires the action of dynamic intracellular tubular structures that structurally and functionally resemble contractile phage tail sheaths [7]. It was concluded that such structures form the secretion machinery and, in addition, that contraction of the T6SS sheath provides the energy needed to translocate proteins [7].

Based on the conserved proteins of T6SS, such as the secreted VgrG and Hcp proteins, homologues of the T4 phage needle complex and a phage tail tube protein respectively, and VipA and VipB, which form tubuli with resemblance to the T4 contracted tail sheath, the secretion system can be divided into four or five major phylogenetic groups [1,2]. A lone member of one of these groups, and a phylogenetic outlier, is the T6SS of *F*. *tularensis*, a highly virulent Gram-negative intracellular pathogen, which causes the zoonotic disease tularemia in humans and many mammals [8]. The T6SS is encoded by a 33-kb gene cluster, the *Francisella* pathogenicity island (FPI), which comprises 17-20 genes that form a secretion system that secretes up to 8 FPI-encoded substrates during intramacrophage infection [9-11]. Studies on FPI mutants have revealed that bacteria replicate only after phagosomal escape and, thus, mutants that are incapable of escape show a null phenotype with lack of intracellular growth, no cytopathogenic effects, and avirulence in experimental models [12-19]. In addition, uptake of *F*. *tularensis* bacteria leads to rapid induction of a proinflammatory response, which is repressed upon bacterial internalization via modulation of host cell signaling and, again, execution of these mechanisms appears to require a cytosolic localization of bacteria [17,19-22]. A majority of FPI mutants have shown dichotomous phenotypes also in this respect and the mutants that are unable to escape from the phagosome do not repress of host cell signaling, whereas other mutants show the same phenotypes as the parental strains [19,22]. Two notable exceptions are the Δ*iglI* and Δ*iglG* mutants of LVS, since these are avirulent but show intact growth in certain monocytic cells, although with only marginal cytopathogenic effects [17].

An FPI protein of special interest is PdpC, since a truncated form of the protein has been identified in FSC043, an attenuated, spontaneous mutant of the prototypic *F*. *tularensis* subspecies *tularensis* strain SCHU S4 [23]. We have previously characterized the FSC043 strain and observed that it displays impaired replication in murine monocytic cells [24]. Therefore, we hypothesized that the spontaneous mutation could be related to the impaired intracellular replication of the mutant. In the present study, we generated and characterized a Δ*pdpC* mutant of *F*. *tularensis* LVS. We observed a phenotype that was distinct from all previously described FPI mutants, since it showed very impaired phagosomal escape and lack of intramacrophage replication, but still pronounced cytopathogenic effects, although distinct from those of the parental strain.

## Results

### In *silico* analyses and localization of PdpC

**Figure 1:**
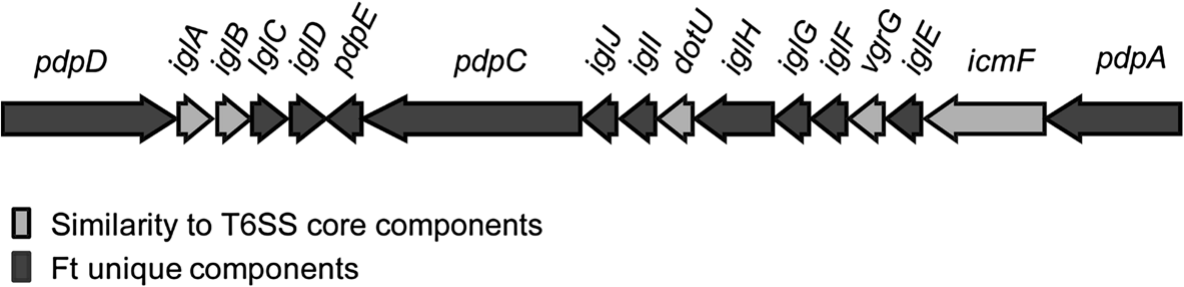
**The *Francisella *pathogenicity island. **The two operons are depicted as a sequence of arrows in the direction of transcription. Arrows in light grey indicate genes with homology to known T6SS core components, while arrows in dark grey represent genes that lack T6SS component homology.

To characterize PdpC, in *silico* analyses together with cell fractionation were carried out. PdpC was predicted to be a cytoplasmic 156-kDa protein with putative transmembrane regions. Standard algorithms did not identify any conserved domain, signal peptide, lipoprotein signal, or secretion signal, and no homologies to non-*Francisella* proteins were found. Homologues exist in all species and subspecies of *Francisella*, however, they are not identical to PdpC of LVS. For example, PdpC in both LVS and SCHU S4 contains 1,328 amino acids, whereas the *F*. *novicida* U112 homologue contains 1,325 amino acids. The former two show only 18 amino acid differences, whereas 71 and 72 amino acids (95% identity), respectively, are distinct compared to the *F*. *novicida* variant. Figure 1 shows a representation of the different genes found in the FPI, and the localization of *pdpC* at the end of one of the two putative operons.

**Figure 2:**
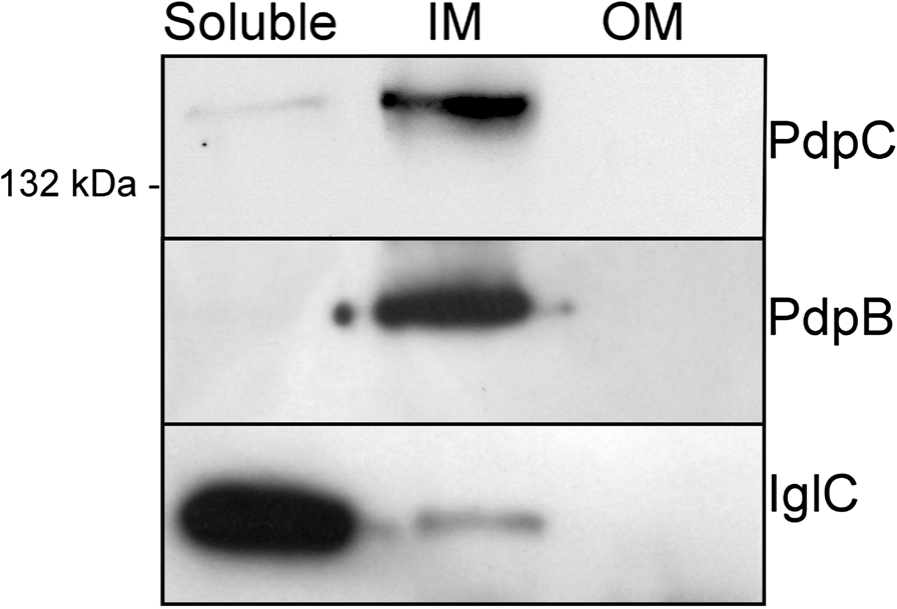
**Subcellular localization of PdpC. **LVS whole-cell lysate was separated into soluble, inner membrane (IM) and outer membrane (OM) fractions using ultracentrifugation and Sarkosyl treatment. After separation by SDS-PAGE, the presence of PdpC in each fraction was determined by Western blot using polyclonal anti-PdpC antibodies. Antibodies recognizing IglC and PdpB were used as markers for soluble and inner membrane fractions, respectively.

To investigate the subcellular localization of PdpC, LVS bacteria were separated into soluble, inner membrane, and outer membrane fractions and the amounts of the protein in each fraction determined by immunoblot analysis. PdpC was found to be predominantly an inner membrane protein, but a small portion was also found in the soluble fraction (Figure 2). It is likely that the transmembrane regions identified in the *in silico* analysis may contribute to its membrane location.

### Construction and phenotypic characterization of a Δ*pdpC* null mutant

To determine the role of PdpC in *F*. *tularensis* LVS, an in-frame deletion mutant was constructed by deletion of both copies of the gene. To verify the absence of PdpC in the mutant, immunoblot analysis with an anti-PdpC antibody was performed on bacterial pellets and real-time PCR was used to quantify the transcription levels of *pdpC*. No immunoreactive protein or gene transcript was detected in the mutant, whereas expression of the downstream *pdpE* gene was not affected (data not shown and Table 1), indicating that the deletion conferred no polar effect. For complementation in *cis*, the *pdpC* gene was introduced in the original site of one of the pathogenicity islands of the mutant.

**Table 1:** **Differences in FPI mRNA expression between **Δ***pdpC *and LVS**

	**Average**^**a**^	***P *value**
*vgrG*	−1.49	0.176
*iglH*	−3.09	0.119
*pdpC*	−6.8 × 10^-6^	<0.001
*pdpE*	−0.26	0.913
*iglD*	−3.46	0.010
*iglC*	−3.99	0.055
*iglB*	−2.97	0.040
*iglA*	−3.75	0.080

^a ^The results show the average fold change in gene expression from 7 experiments. The results for the other FPI genes indicated no significant changes in the mRNA expression between Δ*pdpC *and LVS.

A Student’s *t*-test was used to determine if the difference in fold change was significant between LVS and the Δ*pdpC *mutant.

Since PdpC was found to localize to the bacterial inner membrane, it would be possible that its absence affected the integrity of the bacterial membrane and, therefore, we investigated whether Δ*pdpC* may be defective for membrane integrity and/or sensitive to stress stimuli. We found this particularly pertinent in view of the recent finding that so called hypercytotoxic *F*. *tularensis* mutants, often deficient for membrane-associated proteins or LPS, are prone to intracellular lysis, which leads to increased levels of pyroptosis [25]. The LPS profile of Δ*pdpC*, as judged by use of an LPS antibody, was indistinguishable from that of LVS (data not shown) and, moreover, it did not show increased susceptibility to a detergent, SDS, a cell-permeable dye, EtBr, or an antibiotic that penetrates deficient Gram-negative membrane, Vancomycin, nor to stress-related stimuli such as low pH, temperature, or H_2_O_2_ (Additional file 1: Table S1). Additionally, since it was shown that growth of hypercytotoxic mutants was delayed in Chamberlain’s medium, but not in TSB [25], *in vitro* growth of the Δ*pdpC* mutant was investigated. However, the mutant grew as well as LVS in both Chamberlain’s medium and TSB as well as on solid media. Therefore, we conclude that the Δ*pdpC* mutant showed intact membrane integrity and thereby none of the features typical of hypercytotoxic mutants.

**Figure 3:**
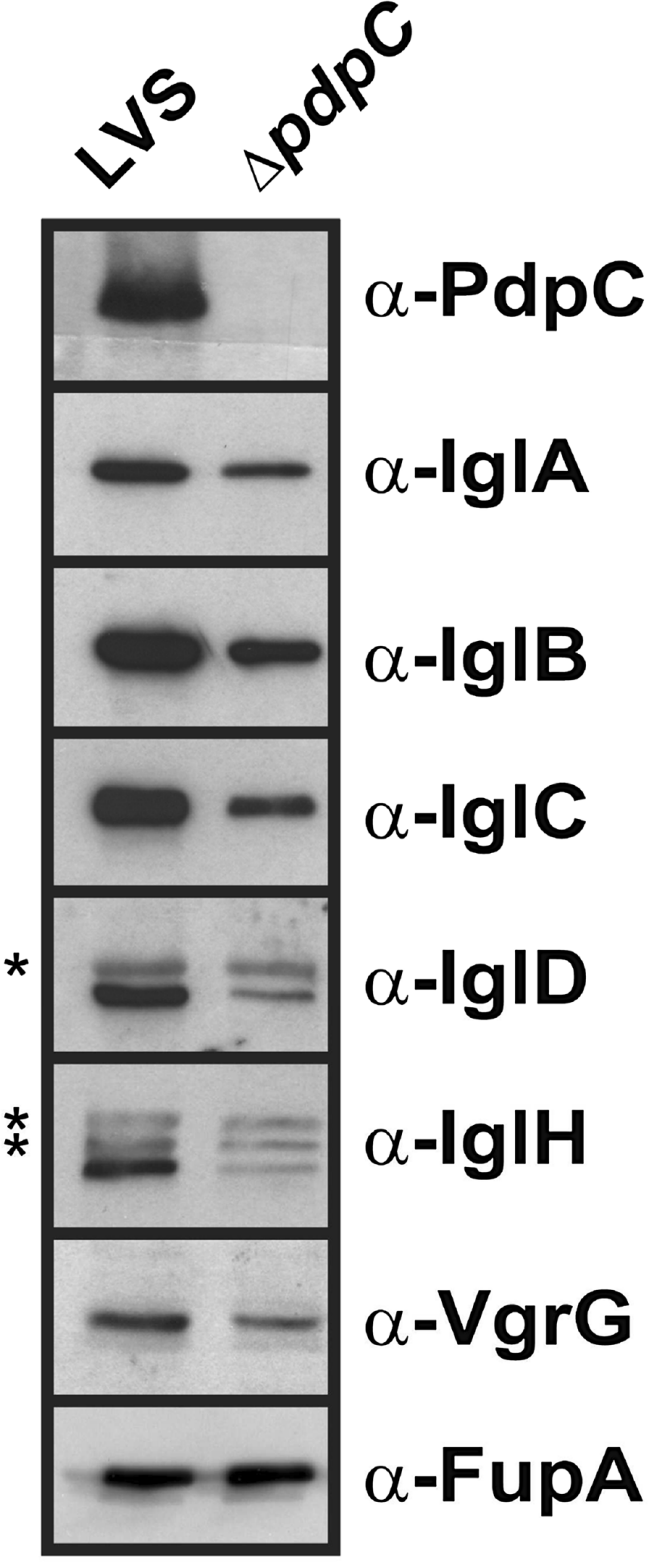
**Western blot analysis comparing the levels of FPI proteins between LVS and the **Δ***pdpC *mutant. **Whole-cell lysates of *Francisella *were separated on SDS-PAGE and FPI protein-specific antibodies were used to detect the levels of proteins in the two samples. An antibody against FupA was used as a loading control. Asterisks indicate unspecific bands. The assay was repeated at least three times.

By performing PCR using primers specific for *pdpC* and other FPI genes, we found that *pdpC* was part of a large transcript including the 12 FPI genes from *pdpA* to *pdpE* (data not shown). To investigate the possibility of polar effects in the mutant, we measured the expression of FPI genes using RT-qPCR. The transcription of genes directly upstream of *pdpC* was not affected, nor was there any effect on the *pdpE* gene immediately downstream, indicating a lack of polar effects of the gene deletion, while, surprisingly, the genes in the *iglAD* operon were downregulated, although only two of them to a significant extent (Table 1). The downregulation also included the corresponding proteins, IglA, B, C, and D, but also the levels of VgrG and IglH were lower in the mutant (Figure 3). Thus, there appear to be both transcriptional and translational effects resulting from the absence of PdpC. The absence of *pdpC* did not affect expression of any of *mglA*, *sspA*, *pmrA* genes (data not shown), all of which encode proteins that positively regulate FPI expression [26]. We also used a bacterial two-hybrid (B2H) assay to determine the possibility that PdpC may form a regulatory complex together with the FPI regulatory proteins SspA, MglA, FevR, and PmrA [9], but none of these were found to interact with PdpC, although a novel PmrA-PmrA interaction was determined, nor did PdpC interact with any of the other members of the FPI (data not shown). Thus, PdpC does not appear to be part of a positive transcriptional regulatory complex, but affects *iglABCD* expression by an unknown mechanism.

### The Δ*pdpC* mutant shows a distinct form of phagosomal escape

**Figure 4:**
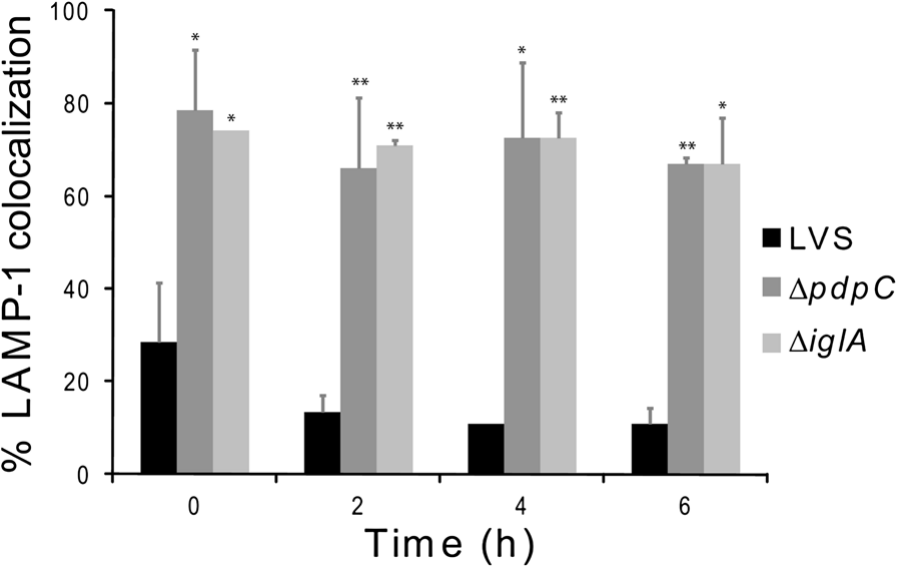
**Phagosomal escape of *F. tularensis. *Colocalization of GFP**-**expressing *F. tularensis *strains and LAMP-1. **J774 cells were infected for 2 h with *F*. *tularensis* strains expressing GFP (Green fluorescent protein) and, after washing, incubated for indicated time points. Fixed specimens were labeled for the late endosomal and lysosomal marker LAMP-1. 100 bacteria were scored per sample and time point. Results from a representative experiment are shown. Bars represent mean values and error bars are used to indicate standard deviations. Asterisks indicate that the colocalization differs significantly from that of LVS (*: *P* < 0.05; **: *P* < 0.01).

**Figure 5:**
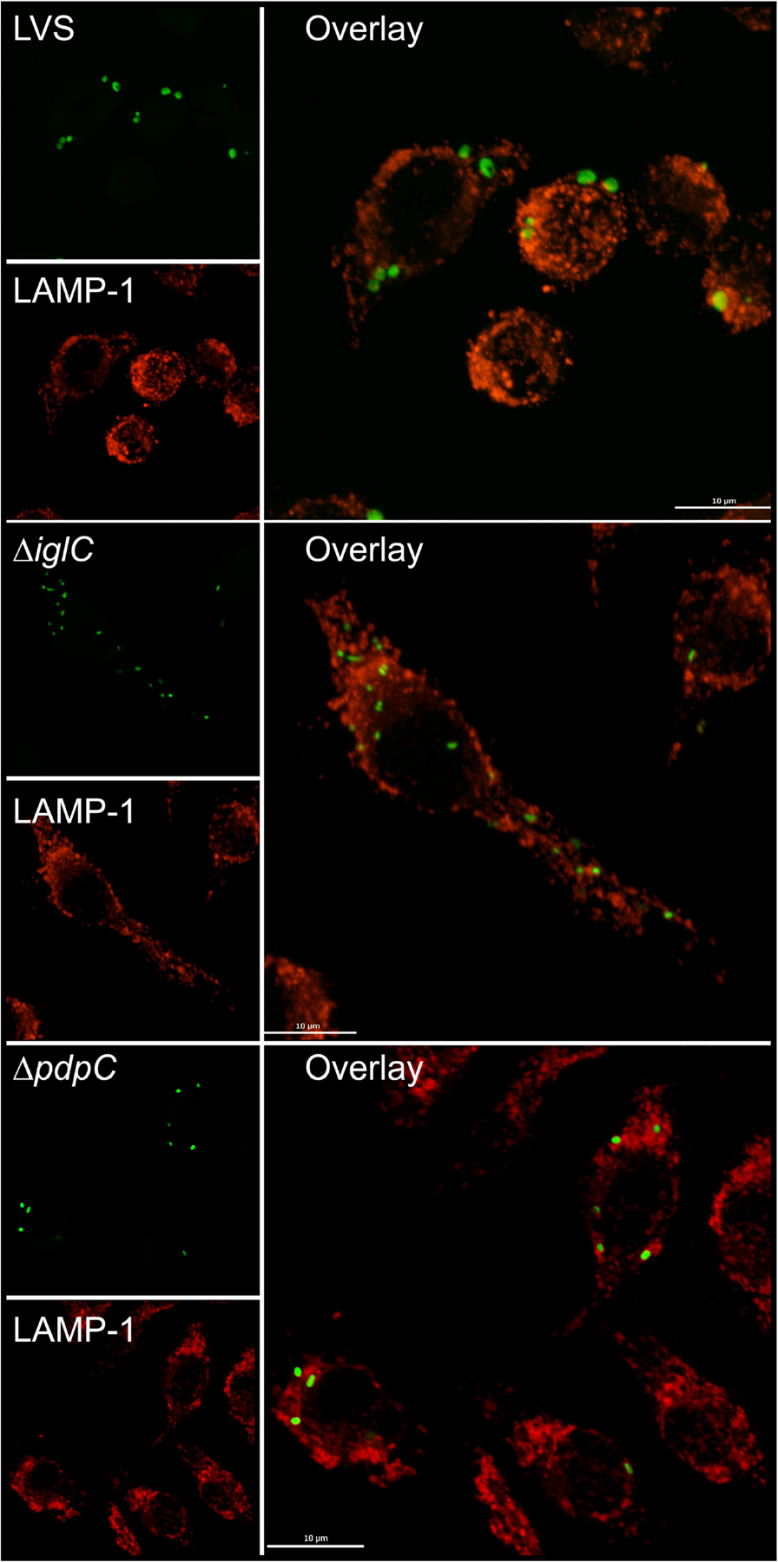
**Colocalization of GFP-expressing *F. tularensis *strains and LAMP-1. **J774 cells were infected with the LVS, the Δ*pdpC *mutant, or the Δ*iglC *mutant expressing GFP (Green fluorescent protein) at an MOI of 200 and, after washing, incubated for 6 h. Colocalization of GFP-labeled *F*. *tularensis *and LAMP-1 on fixed and labeled specimens was analyzed using a confocal microscope (Nikon Eclipse 90i, Nikon, Japan). Scale bar 10 μm.

**Figure 6:**
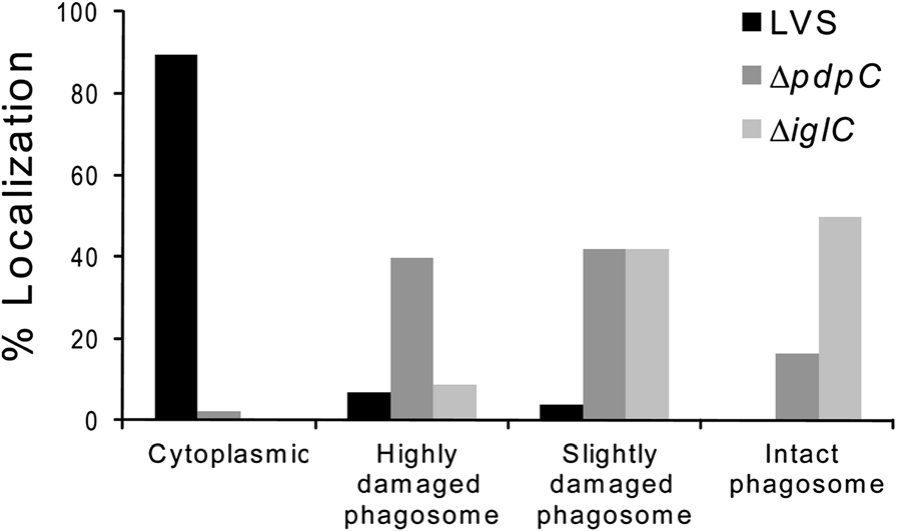
**Subcellular colocalization in J774 cells of *F. tularensis *bacteria. **J774 cells were infected for 2 h with *F*. *tularensis *strains and, after washing, incubated for 6 h. Bacteria were examined using transmission electron microscopy (TEM) and categorized into one of four categories depending on the preservation of the phagosomal membrane. At least 100 bacteria per sample were scored. Results from a representative experiment are shown.

**Figure 7:**
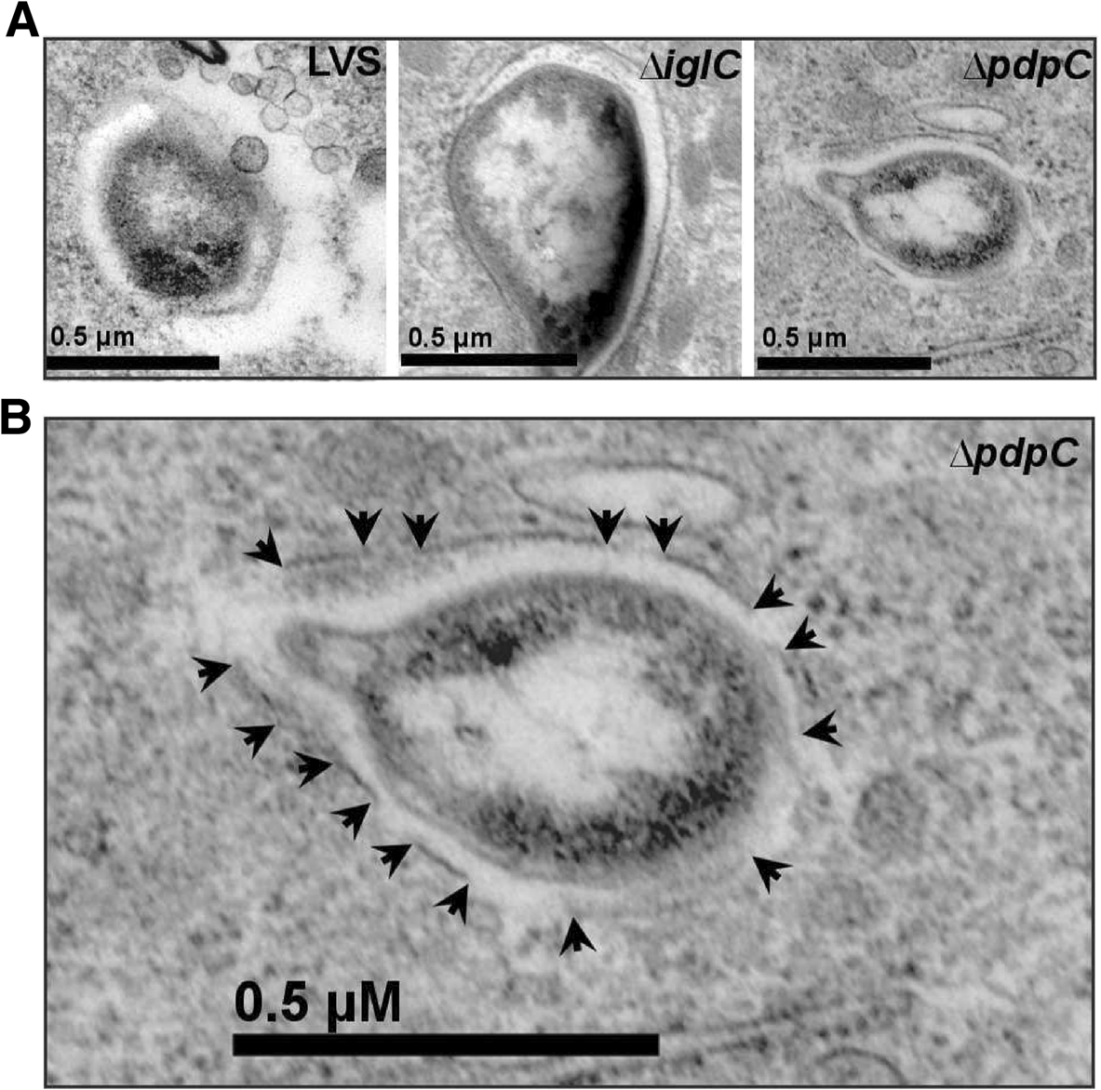
**Electron micrographs of J774 macrophages infected with *F. tularensis. ***(**A**) Cells infected with LVS, the Δ*pdpC *mutant, or the Δ*iglC* mutant. (**B**) A close-up of the Δ*pdpC *micrograph from A. Black arrows indicate the borders of the remaining vacuolar membranes surrounding the intracellular bacterium.

Previous studies have demonstrated that many of the FPI genes are directly or indirectly necessary for the phagosomal escape (reviewed in [9]). Often the subcellular localization is determined by antibodies against LAMP-1, a marker of late endosomes or lysosomes acquired within 30 min after uptake of *F*. *tularensis* (reviewed in [27]). Therefore, confocal microscopy was used to determine the percentage of LAMP-1 that colocalized with Green fluorescent protein (GFP)-expressing Δ*pdpC* in J774 macrophages up to 6 h. At this time point, we have previously observed that essentially all LVS bacteria had escaped from the phagosome [17] and this was confirmed in the present study since only 10.8 ± 3.5% colocalized with LAMP-1, while the corresponding numbers for Δ*iglA*, the negative control, were 67.0 ± 9.9% (*P* < 0.05 vs. LVS) (Figures 4 and 5). For the Δ*pdpC* mutant, the numbers were 67.0 ± 1.4% (*P* < 0.01 vs. LVS), suggesting that the mutant, similar to Δ*iglA*, does not escape from the phagosome (Figures 4 and 5). Even at 16 and 24 h, the percentages of LAMP-1-colocalized bacteria were around 70% for Δ*pdpC* (data not shown). To further investigate the intracellular localization of the mutant, transmission electron microscopy (TEM) was performed. J774 cells were infected with LVS, Δ*pdpC* or Δ*iglC*, and the percentage of cytosolically located bacteria determined. At 6 h, as many as 89.3% of the LVS bacteria were found free in the cytoplasm while a small population, 10.7%, was surrounded by highly damaged (< 50% of membranes intact) vacuolar membranes (Figures 6 and 7). At the same time point, 50% of the Δ*iglC* mutant bacteria were surrounded by intact vacuolar membranes, 42% by slightly damaged vacuolar membranes (> 50% of membrane intact), whereas only ~ 15% of the vacuolar membranes were intact around the Δ*pdpC* bacteria and ~40% of membranes were slightly damaged and 40% highly damaged (Figures 6 and 7). This suggests that Δ*pdpC*, in contrast to the Δ*iglC* mutant, clearly affected the preservation of the phagosomal membranes. At 18 h the majority, 96%, of the LVS bacteria were found free in the cytoplasm, whereas a majority of the Δ*pdpC* bacteria still co-localized to highly damaged, 45%, or slightly damaged vacuolar membranes, 28%. Around 25% of the Δ*pdpC* bacteria were surrounded by membranes that were less than 10% intact, however, even these bacteria were distinct from the LVS bacteria, since they were not completely free in the cytosol.

These findings appeared to be contradictory, since the LAMP-1 colocalization data suggested that the degree of phagosomal escape of Δ*pdpC* was similar to the Δ*iglA* and Δ*iglC* mutants, prototypes for the phagosomally located mutants, whereas the TEM data indicated distinct differences between the Δ*iglC* and Δ*pdpC* mutants. We believe that the findings can be reconciled, however, since the TEM data indicated that essentially no Δ*pdpC* bacteria were free in the cytoplasm, whereas ~ 80% were surrounded by slightly or highly damaged membranes. This unusual phenotype demonstrated that a majority of the Δ*pdpC* bacteria was closely adjacent to membrane parts, in agreement with the confocal microscopy data indicating that 60-75% of the bacteria colocalized with LAMP-1. Therefore, the mutant will show a high percentage of colocalization although not being confined to an intact phagosome. Thus, we conclude that PdpC directly or indirectly plays a very important role for the normal phagosomal escape.

### The Δ*pdpC* mutant shows defective intracellular replication

**Figure 8:**
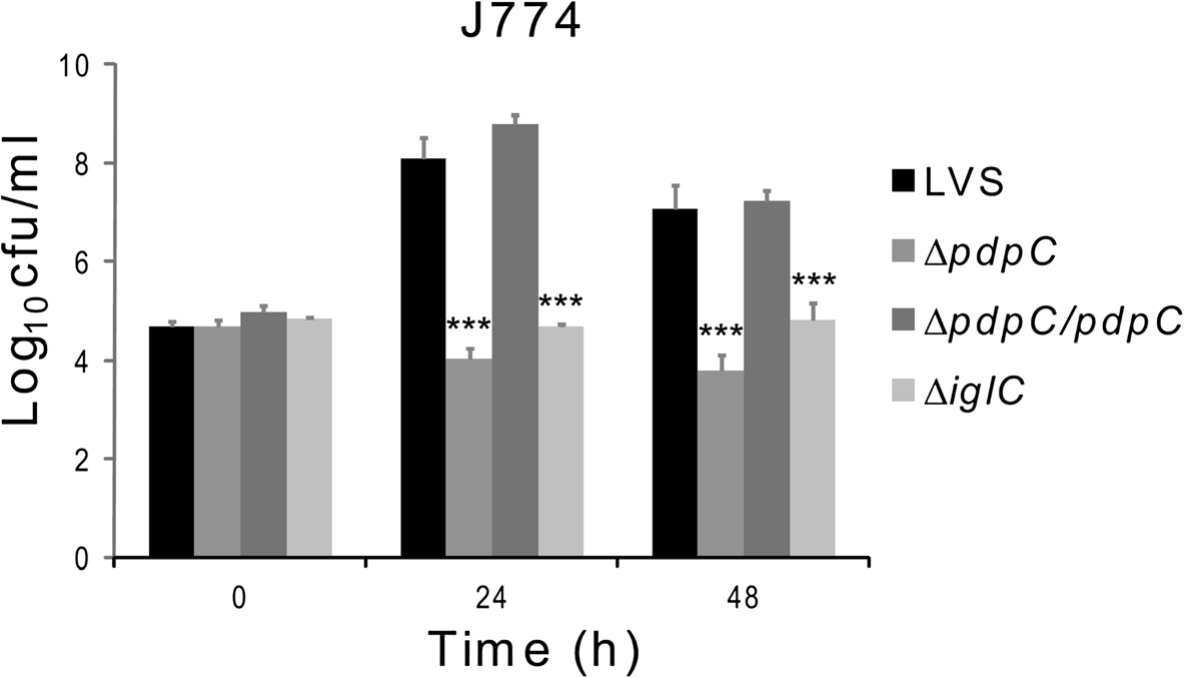
**Intracellular growth of *F. tularensis *in J774 cells. **LVS, the Δ*pdpC *mutant, the complemented Δ*pdpC* mutant, or the Δ*iglC *mutant was used to infect J774 cells for 2 h, after which the monolayers were washed and medium added (corresponds to 0 h). Cells were then incubated for 24 or 48 h before being lysed, serially diluted and plated to estimate the number of CFU. The graph presents a representative experiment out of eight performed. Each bar represents the mean value and the error bars represent the standard deviation. The asterisk indicates that the log_10 _data differs significantly from LVS (***: *P* < 0.001). Two-sided Student’s *t*-test was used to compare means.

To determine whether the partial degradation of phagosomal membranes observed for Δ*pdpC* was sufficient to allow intracellular growth, murine macrophage-like J774 cells were infected with LVS, the Δ*pdpC* mutant, or the complemented strain. The number of bacteria at time 0 was identical for the LVS strain and the Δ*pdpC* derivatives in all experiments performed, so the distinct phenotypes of the mutant could not be explained by differences in its uptake by phagocytes. While LVS and the complemented strain replicated approximately three log_10_ CFU within the first 24 h, the Δ*pdpC* mutant showed no growth (Figure 8). In additional experiments, there were no significant increases in bacterial numbers during the first 6 h, or at 48 or 72 h (data not shown). The results unambiguously demonstrated that the Δ*pdpC* mutant had a markedly impaired ability to replicate intracellularly. Replication was also assessed in BMDM and PEC and the results were similar to those obtained with J774 cells; the mutant showed no replication whereas the complemented strain replicated as well as LVS (data not shown). To further verify the inability of the mutant to grow intracellularly, bacterial RNA was isolated from infected cells and the expression of the *F*. *tularensis* 16S rRNA gene was measured. We observed a 1.4 log_10_ decrease of 16S rRNA in Δ*pdpC*-infected cells during the first 24 h, while LVS infected cells showed an increase of 2.8 log_10_. The data demonstrate that, regardless of method and macrophage type utilized, the Δ*pdpC* mutant showed no significant intracellular replication and the deficient phenotype could be restored by complementation of the mutation in *cis*.

### The Δ*pdpC* mutant shows attenuation *in vivo*

**Figure 9:**
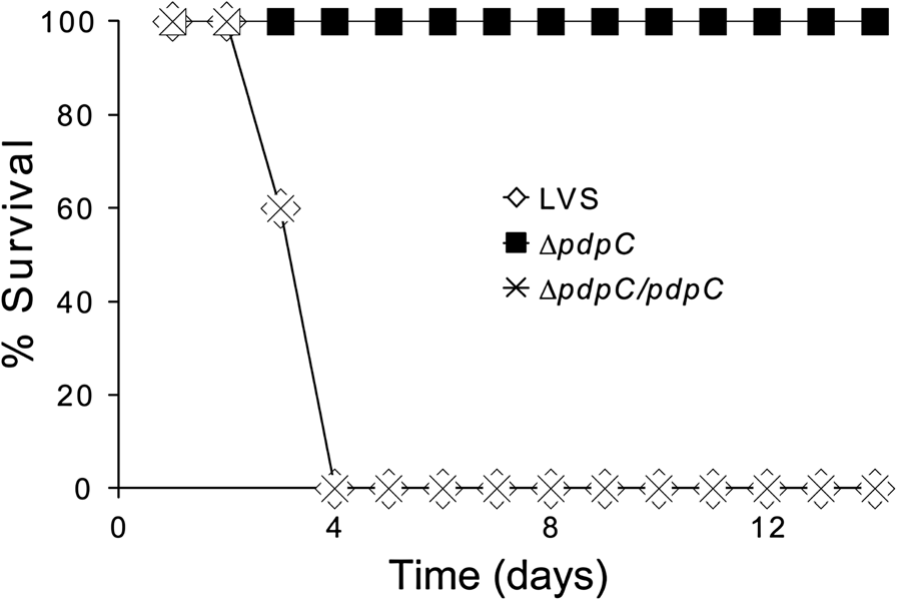
**Survival of C57BL/6 mice after intradermal infection with 5 × 10**^**7**^ **CFU of LVS or the complemented Δ*pdpC *mutant, or 1 × 10**^**9**^ **CFU of the Δ*pdpC *mutant (5 mice/group). **All mice of the latter group survived until the experiment was terminated after 28 days.

The lack of intracellular replication observed for the Δ*pdpC* mutant suggested that it is likely attenuated *in vivo*. To test this, mice were infected by the intradermal route with LVS, the Δ*pdpC* mutant, or the complemented mutant. The model has been widely used [25,28-32] and identifies even marginal levels of attenuation since the LD_50_ for LVS is estimated to be approximately 2 × 10^7^ CFU [33]. With an infection dose of 4 × 10^7^ CFU, LVS caused 80% mortality (mean time to death 4.3 ± 0.5 days) and all mice infected with the complemented strain died within 4 days (mean time to death 3.6 ± 0.5 days). In contrast, only two mice died after infection with a 20-fold higher dose, 8 × 10^8^ CFU, of Δ*pdpC* (mean time to death 3.0 ± 0.0 days). In a second experiment, all mice died within 4 days when infected with a dose of 5 × 10^7^ CFU with LVS or the complemented strain, whereas no mice died after infection with a dose of 1 × 10^9^ CFU of the Δ*pdpC* mutant (Figure 9). Thus, PdpC directly or indirectly plays a very critical role for the virulence of *F*. *tularensis*. To determine the bacterial burden in organs, spleens were isolated 5 days after infection with a dose of 3 × 10^2^ CFU of LVS or the Δ*pdpC* mutant and 16 days after infection with 1 × 10^7^ CFU of either strain. In the latter experiment, three out of five LVS infected mice died. No bacteria were found in any of the spleens on day 16, whereas both LVS and Δ*pdpC* bacteria were isolated on day 5, the former were 70-fold more numerous, 4.7 log_10_ vs. 2.8 log_10_. Thus, although much attenuated, the Δ*pdpC* mutant was capable of limited systemic spread.

### Δ*pdpC* induces an MOI-dependent cytopathogenic response

**Figure 10:**
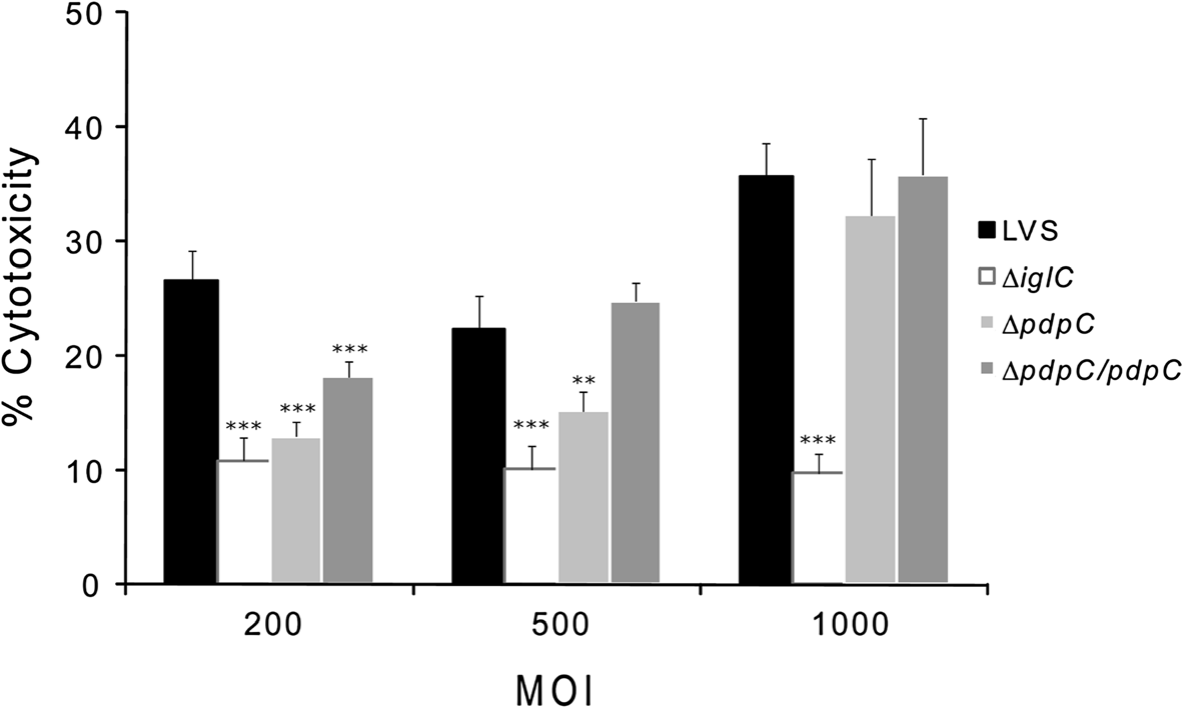
**LDH release from *F.tularensis-*infected cells. **Culture supernatants of infected J774 cells were assayed for LDH activity at 24 h with a MOI of 200, 500, or 1,000. The activity was expressed as a percentage of the level of uninfected lysed cells. The value of uninfected cells at 24 h was 14.6 ± 1.6%. Means and SEM of six replicate wells are shown. The asterisks indicate that the LDH levels were significantly different to those of LVS-infected cells at the same time point as determined by a two-sided *t*-test with equal variance (**: *P* < 0.01, ***: *P* < 0.001).

Previous studies on FPI mutants have revealed a strong correlation between phagosomal escape and cytosolic replication on one hand and cytopathogenicity on the other (reviewed in [9]). The cytopathogenic response resulting from an *F*. *tularensis* infection is characterized by morphological changes such as membrane blebbing, cell detachment, LDH release, and DNA fragmentation [34]. To determine whether Δ*pdpC* induced cytopathogenicity, J774 cells were infected and the release of LDH into the cell culture supernatants measured and morphological effects on the cells were investigated using phase contrast microscopy. In view of the previously published findings that the cytopathogenic effects in most cases correlated to the intracellular replication of the FPI mutants, we reasoned that the MOI could affect the cytopathogenic effect resulting from the Δ*pdpC* infection, although the mutant did not replicate intracellularly. Indeed, with an MOI of 200, the LVS infection resulted in significant release of LDH, but the Δ*pdpC* infection only in low release, at levels comparable to that of Δ*iglC*-infected cells (Figure 10). At an MOI of 500 or 1,000, the LDH levels from LVS- or the complemented Δ*pdpC* mutant-infected cell cultures were similar and much higher than Δ*iglC*-infected cultures (*P* < 0.001), whereas the Δ*pdpC* mutant showed an intermediate value at an MOI of 500 (*P* < 0.01 vs. LVS) and as high as LVS at the highest MOI (Figure 10). Regardless of the MOI, there was no intracellular growth of Δ*pdpC* recorded (data not shown). Thus, infection with the Δ*pdpC* mutant leads to significant and MOI-dependent cytopathogenic effects despite its lack of intracellular replication. This phenotype is distinct from that of LVS, but also from bacteria located inside intact phagosomes, such as Δ*iglC*.

### Modulation of macrophage inflammatory responses by the Δ*pdpC* mutant

Previous studies have identified an active suppression by *F*. *tularensis* on the ability of host cells to secrete TNF-α in response to *E*. *coli* LPS, an inflammasome-independent process [21,35]. Mutants confined to the phagosome lack this suppressive property [17,19,35]. To characterize the effects of the Δ*pdpC* mutant, J774 cells were infected and cell culture supernatants were analyzed for the presence of TNF-α after 120 min of LPS-stimulation. Efficient and comparable inhibition of TNF-α release was observed after infection with LVS and Δ*pdpC*, but not after infection with the control strain Δ*iglA* (Table 2). Thus, the phenotype of the Δ*pdpC* mutant is clearly distinct from that of bacteria enclosed in intact phagosomes.

**Table 2:** **TNF**-**α secretion of LPS**-**stimulated J774 cells infected with *F***. ***tularensis***

**Strain**	**TNF-α secretion (pg/ml)**^**a**^
-	708 ± 102
LVS	45.9 ± 8.9^***^
Δ*pdpC*	36.4 ± 7.5^***^
Δ*iglA*	1340 ± 126

^a^*F*. *tularensis*-infected, or uninfected (-), J774 cells were incubated for 2 h with LPS. The average TNF-α secretion in pg/ml with standard errors of triplicate samples is shown, results are from one representative experiment out of three. A Student’s *t*-test revealed that there was no significant difference in TNF-α secretion between LVS and Δ*pdpC *mutant infected cells, but that cells infected with either strain had a significantly lower TNF-α secretion than uninfected cells (^***^: *P* < 0.001).

The rapid phagosomal escape of *F*. *tularensis* into the macrophage cytosol is critical for the efficient inflammasome-dependent induction of IL-1β secretion [17,20,22,36-38]. As a result, mutants with no or delayed phagosomal escape, *e*.*g*., Δ*iglA*, Δ*iglC*, Δ*iglG*, Δ*iglI*, Δ*dotU*, or Δ*vgrG*, exhibit no or very diminished IL-1β release [17,19,22,38]. The cytokine was measured in supernatants of BMDM infected with LVS, Δ*pdpC*, the complemented Δ*pdpC* mutant, or the control strain Δ*iglC* at 5 or 24 h. In supernatants from LVS-, complemented Δ*pdpC*-, and Δ*pdpC*-infected cell cultures, levels were low or below the detection level of the assay at 5 h, but much higher at 24 h, especially for the LVS- and the complemented Δ*pdpC*-infected cultures, whereas levels were below the detection level of the assay for Δ*iglC*-infected cultures or uninfected cells regardless of time point (Table 3). Thus, Δ*pdpC* demonstrated an intermediate phenotype with regard to the IL-1β release, consistent with the incomplete cytosolic escape of Δ*pdpC*, whereas the complemented mutant showed a phenotype indistinguishable from LVS. These data support the notion that inflammasome activation does not occur when the bacteria are confined to intact phagosomes, whereas even the partial disruption of the phagosomal membranes, as executed by Δ*pdpC*, leads to highly significant, but intermediate levels inflammasome-activating cytosolic signaling. This is a slightly modified hypothesis compared to the previously proposed, suggesting that there was a direct correlation between cytosolic location and inflammasome activation [17,20,22,38].

**Table 3:** **IL**-**1β secretion from *F***. ***tularensis***-**infected BMDM cells**

**Strain**	**IL-1β secretion (pg/ml)**^**a**^	
	**5 h**	**24 h**
-	BDL***	BDL***
LVS	76.3 ± 10.9	497.1 ± 79.0
Δ*iglC*	39.6^b^	BDL***
Δ*pdpC*	64.5 ± 27.2	112.1 ± 41.0*
Δ*pdpC*/*pdpC*	163.2 ± 50.2	506.9 ± 94.3

^a^*F*. *tularensis*-infected, or uninfected (-) BMDM cells were incubated for 5 or 24 h. The average IL-1β secretion in pg/ml with standard errors of triplicate biological samples from one representative experiment, out of three, is shown. A Student’s *t*-test was used to determine if the IL-1β secretion was significantly different between LVS infected and mutant infected cells (*: *P* < 0.05, **: *P* < 0.01, ***: *P* < 0.001). BDL means that the concentration was below the detection limit of the assay (< 31.25 pg/ml).

^b^ Only one of the triplicates was above BDL.

## Discussion

*F*. *tularensis* is capable of rapid escape from the phagosome, which is followed by efficient growth within the cytosol of monocytic cells. The molecular mechanisms behind the intracellular life style of the bacterium are not well understood, but have been shown to be dependent on many FPI-encoded genes, of which the most well-studied are the members of the *iglABCD* operon [16,28,37]. Evidence indicates that many of the FPI proteins collectively constitute a T6SS, however, while such systems have been identified in nearly 100 different bacterial species to date, their homologies to the FPI system are weak, indicating that the latter constitutes an evolutionarily distinct group [1,14,22]. While the FPI proteins IglA, IglB, PdpB, VgrG, and DotU show modest similarities to common components of T6SSs, the remaining FPI proteins appear to be unique and this makes it laborious and tedious to understand their roles and functions. The accumulating evidence indicates that many of them are essential core components and as such critically required and, thereby, their absence leads to a null mutant phenotype characterized by lack of phagosomal escape, no intracellular replication, and avirulence [9]. A majority of the investigated FPI mutants appears to belong to this group but, in contrast, the Δ*pdpE* mutant exhibits full virulence [17]. Not all of the investigated mutants so far fit into this polarized pattern, however, since the Δ*iglI* and Δ*iglG* mutants of LVS show delayed cytopathogenicity and a lack of virulence, although intact intracellular replication in some macrophage types [17].

In a recent study, we characterized the markedly attenuated FSC043 strain, a spontaneous mutant of the highly virulent strain SCHU S4, belonging to subspecies *tularensis*. Whole-genome sequencing revealed that only one deletion event and three point mutations discriminated the strains, two of which were identical single nucleotide deletions in each of the two copies of *pdpC*[23]. Although one of the other mutated genes was *fupA*, which confers the most important contribution to the attenuation of LVS, we observed other features of the FSC043 strain that were distinct from those observed for a Δ*fupA* mutant and this led to our interest in understanding the role of PdpC [24]. The present investigation reveals that the Δ*pdpC* mutant of LVS is another example of an FPI mutant with a very distinct and paradoxical phenotype, since it in some aspects mimics that of the LVS strain, whereas it in other aspects is very different since it does not fully escape into the cytosol, lacks intramacrophage replication, and is highly attenuated in the mouse model.

*F*. *novicida* strain U112 has been widely used to study the functions of the FPI, presumably since it harbors only one copy of the FPI and, thus, is more amenable to genetic manipulation and, moreover, does not require BSL3 containment. However, the results are not always in agreement when FPI mutants of *F*. *tularensis* and *F*. *novicida* are studied, as exemplified by our recent finding that *iglI* mutants of *F*. *novicida* and LVS show distinct phenotypes [17]. Moreover, a recent study of *F*. *novicida* FPI mutants revealed that a Δ*pdpC* mutant showed normal intracellular replication in murine cells and also in insect cells and *Drosophila melanogaster*[39-41]. Our only explanation for the disparate results on the Δ*pdpC* mutants is that the functions of PdpC are distinct between the U112 strain of *F*. *novicida* and the LVS strain. In support of this hypothesis, there are 72 amino acids that discriminate the two proteins.

In view of the paradoxical phenotypes of Δ*pdpC*; lack of intracellular replication, but much more distinct cytopathogenic effects than the Δ*iglC* mutant, to some extent resembling those of the so called hypercytotoxic mutants that were recently identified by Peng et al. [25], we found an in-depth analysis of the physical properties of the mutant warranted. An additional rationale was that our bacterial fractionation assay revealed that PdpC predominantly is an inner membrane protein and the hypercytotoxic phenotype has been suggested to be caused by physical instability of mutants that, not surprisingly, are defective for important membrane proteins, or components of the LPS or O-antigens [25,42]. This instability leads to bacterial lysis in the cytosol, which normally does not occur for the LVS or U112 strains. However, regardless of methodology used, we did not observe any similarity between the Δ*pdpC* mutant and the hypercytotoxic mutants and we therefore believe that the phenotype of the former depends directly or indirectly on the functions of PdpC and not on any physical instability of the mutant. The distinct expression of FPI proteins in the mutant was of interest in this regard, since the IglA, IglB, IglC, IglD, IglH, and VgrG proteins showed markedly lower expression and this was also reflected in lower transcription of the *iglABCD* operon. As most of these proteins play key roles for the virulence of the bacterium, their reduced expression may be important for the distinct phenotype of the mutant and, thereby, the contribution of PdpC to this phenotype may be indirect. One possible mechanism whereby such effects on protein levels could be mediated is via direct protein-protein interactions, however, our two-hybrid analysis revealed no such interaction for PdpC to any other FPI protein nor to any of the FPI regulatory proteins MglA, SspA, FevR, and PmrA. This indicates that one of the roles of PdpC is likely regulatory, but distinct from the MglA/SspA/FevR regulatory complex since this complex affects expression of all FPI proteins.

The findings on the Δ*pdpC* mutant illustrate certain caveats concerning methods to discern the intracellular localization of bacteria. A very widely used assay is based on the late endosomal and phagosomal marker LAMP-1, however, in the case of the Δ*pdpC* mutant, we conclude that the 75% co-localization we observed is not indicative of normal phagosomal entrapment, since the TEM analysis clearly indicated that almost all bacteria were surrounded by slightly or highly damaged membranes, thereby explaining the high degree of LAMP-1 colocalization. This phenotype was very distinct compared to the Δ*iglC* mutant, which was associated almost exclusively with intact membranes at similar time points.

The lack of intramacrophage replication was, not surprisingly, also reflected in a much attenuated phenotype in the mouse model, though the mutant was capable of limited systemic spread. However, the most paradoxical phenotype was that, despite its lack of intracellular replication, the mutant modulated the inflammatory response of the host cells in a way that was different from that of the Δ*iglC* mutant. An assay that clearly illustrates this distinction is secretion of IL-1β. We and others have shown that phagosomally contained mutants, *e*.*g*., Δ*iglC*, do not induce release of this cytokine [17,19,20,22,38], however, the Δ*pdpC* mutant showed much higher levels than Δ*iglC*. This indicates that the damage of the phagosomal membrane is a major trigger for the inflammasome activation. In view of the hypothesis by Peng et al., that the phenotype of the hypercytotoxic mutants is dependent on bacterial lysis in the cytosol [25], which does not occur for wild-type strains, our present data suggest that the lysis of the physically intact bacteria occurs in the phagosome and that the DNA that activates the AIM2 inflammasome is released when the phagosomal membrane is damaged, as is the case for the Δ*pdpC* mutant, but not for the Δ*iglC* mutant. This would explain the intermediate levels of IL-1β secretion induced by the Δ*pdpC* mutant. Another example of the potent immunomodulating effect of the Δ*pdpC* mutant was suppression of the *E*. *coli* LPS-induced TNF-α secretion, an inflammasome-independent event. We have previously concluded that there is a close relationship between the mitigation of the LPS-induced inflammatory response and the subcellular localization of *F*. *tularensis*[17]. The Δ*pdpC* mutant adds to the understanding of this mechanism, since it, as the LVS strain, completely abrogated the TNF-α secretion. Thus, this phenotype is not related to intracellular replication, but only to the ability to disrupt the phagosomal membrane.

The findings reported herein demonstrate that the relationship between bacterial intracellular location and infection-mediated effects on host cell is not always straightforward and indicate that a key event in mediating the latter is the disruption of the phagosomal membrane and presumably the concomitant release of bacterial DNA and effector proteins of the T6SS and possibly other secretion systems. This situation is to some degree analogous to recently published data on mycobacteria. Although *Mycobacterium tuberculosis* and other mycobacteria are primarily considered to be vacuolar pathogens, it has become evident that the ESX-1 secretion system effectuates limited perforation of the phagosomal membrane, although the bacterium still remains within the phagosome. Recent publications demonstrate that this perforation results in mixing of phagosomal and cytoplasmic contents and induces a cytosolic host response triggered by bacterial DNA [43-45]. Thus, although the ultrastructural findings on the Δ*pdpC* mutant are distinct from those on mycobacteria, the bacteria-induced effects on the host cells are in both cases critically dependent on the permeabilization of the phagosomal membranes and leakage of DNA and, possibly, bacterial effectors into the cytosol.

Collectively, our data show that the Δ*pdpC* mutant distinctly modulates the interaction between *F*. *tularensis* and the phagocytic cell, since it shows incomplete phagosomal escape, lack of intramacrophage growth, intermediate cytopathogenic effects, and marked attenuation *in vivo*, but almost intact modulation of the macrophage inflammatory response. The unique phenotype of the mutant provides novel information, since it demonstrates that some of the cytopathogenic effects and modulation of host cell signaling is not dependent on bacterial replication, but only requires disruption of the phagosomal membrane. Therefore, further elucidation of the exact functions of PdpC will be important in order to understand the enigmatic mechanisms behind the intracellular life style of *F*. *tularensis*.

## Conclusions

The pathogenicity of *F*. *tularensis* is intimately linked to expression of the type VI secretion system encoded by the FPI. Our characterization of the FPI mutant Δ*pdpC* demonstrates that is exhibits a unique phenotype compared to other FPI mutants since it exhibited lack of intracellular replication, incomplete phagosomal escape, and marked attenuation in the mouse model, but still efficiently triggered secretion of IL-1β and markedly induced LDH release. The findings implicate that a thorough understanding of the function of PdpC will provide important understanding behind the unique intracellular life cycle of *F*. *tularensis*.

## Methods

### Bacterial strains, plasmids, and growth conditions

Bacterial strains and plasmids used are listed in Additional file 1: Table S2. *Escherichia coli* strains were grown either in Luria Bertani broth (LB) or on Luria agar plates (LA) at 37°C. *F*. *tularensis* was cultured either in Chamberlain’s medium [46] or in TSB at 37°C, 200 rpm, or on modified GC-agar at 37°C, 5% CO_2_. When required, kanamycin (50 μg/ml for *E*. *coli* or 10 μg/ml for *F*. *tularensis*), carbenicillin (100 μg/ml), tetracycline (10 μg/ml), polymyxin B (50 μg/ml) or chloramphenicol (25 μg/ml for *E*. *coli*, 2.5 μg/ml for *F*. *tularensis*) was added to the medium.

The Δ*iglA* or Δ*iglC* mutants were used as controls for phagosomally located bacteria. Both have previously been characterized in detail by us and others, and their phenotypes are indistinguishable in that they are avirulent and show no phagosomal escape or intramacrophage replication [16,47-49].

### Bioinformatic studies

The bioinformatic analysis was performed using the following web-based tools: PSORTb (http://www.psort.org/psortb/index.html) for prediction of localization, TMPred (http://www.ch.embnet.org/software/TMPRED_form.html) to find putative transmembrane regions, SMART (http://smart.embl-heidelberg.de) and BLAST (http://blast.ncbi.nlm.nih.gov/Blast.cgi) for identifying conserved domains, and CBS prediction servers (http://www.cbs.dtu.dk/services) to find a lipoprotein signal, signal peptides or secretion signals.

### Construction of expression vectors and the bacterial-two-hybrid (B2H) assay

For the bacterial two-hybrid assay, PCR-amplified *iglE*, *iglF*, *iglG*, *iglH*, *iglI*, *iglJ*, *pdpC*, *pdpE*, *iglD*, *pdpA*, *pdpD*, *fevR*, and *pmrA* were initially cloned into the pCR4-TOPO TA cloning vector to facilitate sequencing, and subsequently introduced as *Nde*I/*Not*I fragments into the IPTG-inducible plasmids pACTR-AP-Zif and pBRGPω [50]. For alleles containing intrinsic *Nde*I sites (*iglJ*, *fevR*, *pmrA*), these were mutated by overlap PCR prior to cloning. Since PdpD is significantly truncated by an in-frame stop codon in LVS, we used *F*. *tularensis* subsp. *novicida* U112 as template in the overlap PCR reaction to amplify full-length *pdpD* without its intrinsic *Nde*I site. Primer combinations used to construct the B2H alleles are listed in Additional file 1: Table S3. Plasmids were transferred into *E*. *coli* DH5αF’IQ (Invitrogen AB, Stockholm, Sweden) by electroporation. As the reporter strain for the bacterial two-hybrid experiments, the *E*. *coli* strain KDZif1ΔZ was used. It harbors an F9 episome containing the *lac* promoter-derivative p*lac*Zif1-61 driving expression of a linked *lacZ* reporter gene [51]. Cells were grown with aeration at 37°C in LB supplemented with 0.4 mM IPTG (Isopropyl β-D-1-thiogalactopyranoside), permeabilized with SDS-CHCl_3_ and assayed for β-galactosidase (β-gal) activity as described previously [52]. Assays were performed at least three times in duplicate on separate occasions.

### Construction of the Δ*pdpC* null mutant in *F*. *tularensis* LVS and complementation in *cis*

The LVS Δ*pdpC* strain was generated by allelic replacement essentially as described [53]. In brief, the fragments located upstream or downstream of the gene were amplified by PCR and a second overlapping PCR using purified fragments from the first amplification as templates was performed. The PCR fragment was cloned to pDMK3 and the resulting plasmid was first introduced into *E*. *coli* S17-1λpir and then transferred to LVS by conjugation. Clones with plasmids integrated into the LVS chromosome by a single recombination event were selected on plates containing kanamycin and polymyxin B and verified by PCR. Clones with integrations were then subjected to sucrose selection. This procedure selected for a second cross-over event in which the integrated plasmid, encoding *sacB*, was excised from the chromosome. Kanamycin-sensitive, sucrose-resistant clones were examined by PCR confirming the deletion of the gene. The conjugation and further procedures were repeated to remove the second *pdpC* copy. The resulting mutant designated Δ*pdpC* had amino acids 6-1325 deleted in both copies. The *cis* complementation was based on the same procedures, although only the upstream region was amplified together with the *pdpC* gene. This resulting strain with one copy deleted and one wild-type copy restored was designated as Δ*pdpC*/*pdpC*. For both strains generated, PCR and RT-PCR screening was used to verify that the anticipated genetic event had occurred. Primer sequences are listed in Additional file 1: Table S3.

### Western blot analysis

Bacteria were grown on plates, suspended in PBS to OD_600_ 1.0 and the pellet was lysed in Laemmli sample buffer and heated for 10 min to allow full denaturation of proteins. SDS-PAGE was performed and proteins were transferred onto nitrocellulose membranes using a semidry blotter (Bio-Rad laboratories, CA, USA). Membranes were blocked in 5% non-fat dried milk and probed with either mouse monoclonal antibodies recognizing IglB, IglC, or rabbit polyclonal antibodies recognizing IglA (all three from BEI Resources, Manassas, VA, USA), rabbit polyclonal antibodies raised against the specific proteins IglH, VgrG, (Inbiolabs, Tallinn, Estonia), or PdpC (Agrisera, Vännäs, Sweden). Specific chicken IgY was used to detect IglD or FupA, both from Agrisera, Vännäs, Sweden. Secondary antibodies conjugated with horseradish peroxidase (HRP) used were: goat anti-mouse (Santa Cruz Biotechnology, CA, USA), donkey anti-rabbit (GE Healthcare, UK), and rabbit anti-chicken IgY (Sigma-Aldrich, St. Louis, MO, USA). Protein bands were visualized using the Enhanced Chemiluminescence system (ECL) (Amersham Biosciences, Uppsala, Sweden).

### Fractionation of *F*. *tularensis*

Strains were grown in 40 ml Chamberlain’s medium overnight, spun down and resuspended in 5 ml of ice cold TE buffer, followed by sonication to lyse the cells. Intact cells were removed by 30 min of centrifugation (Heraeus, Multifuge 3 S-R, 75006445 swing-out rotor) at 3,450 × *g* at 4°C. The cell lysate was split into soluble and insoluble fractions using ultracentrifugation (Beckman Optima L-80 XP, rotor type SW 41 Ti) for 3 h at 154,000 × *g* at 4°C. The soluble fraction (supernatant) was collected and subjected to centrifugation to remove contaminants (1 h, 154,000 × *g*, 4°C), while the insoluble fraction (membrane pellet), was resuspended in 5 ml of 0.5% Sarkosyl (Sigma) and incubated for 90 min at 4°C while shaking. The pellet fraction was then divided into inner membrane (Sarkosyl-soluble) and outer membrane (Sarkosyl-insoluble) fractions by a second ultracentrifugation step for 3 h at 154,000 × *g* at 4°C. 5 μg of each fraction (protein concentrations were determined using a Nanodrop ND-1000 spectrophotometer (Thermo Fisher Scientific, DE, USA)) was separated by SDS-PAGE followed by transfer to nitrocellulose membrane, and analyzed using standard Western blot techniques (above). Antisera against PdpB/IcmF and IglC, suggested to be IM and soluble proteins respectively [14,54], were used as controls of the purity of the fractions.

### Reverse transcriptase quantitative PCR (RT-qPCR)

Gene expression of various genes was compared between LVS and the Δ*pdpC* mutant grown on agar plates. The details of RNA isolation, DNase treatment, RT-PCR and RT-qPCR have been described elsewhere [18]. No RNA degradation was performed after the RT-PCR. The RT-qPCR reaction was performed using the Power SYBR Green Master Mix (Applied Biosystems) in a 7900HT Sequence Detection System with SDS 2.3 software (Applied Biosystems). The *tul4* gene (FTL0421) was used as a reference gene for normalization after determining that its expression varied minimally between samples. An amplification control was created for each RNA sample by omitting the Reverse Transcriptase during RT-PCR, and a template control was used to confirm that no amplification took place in absence of the cDNA template in the RT-qPCR. Primer efficiency was determined (primers are available upon request), and found to be similar among the primer pairs used, and the 2^-ΔΔCt^ method was used for data analysis. Technical triplicates were loaded for each sample and the experiment was repeated seven times.

### LPS detection

In order to visualize LPS, the outer membrane fraction, see section “Fractionation of *F*. *tularensis*”, was run on SDS-PAGE, and Western blot analysis was performed according to established protocols and a *Francisella* LPS-specific antibody (MAb Fb11) was used to visualize the LPS.

### Multidrug sensitivity assay

The multidrug sensitivity assay was adapted from Gil and colleagues [36]. *F*. *tularensis* strains grown on modified GC-agar base were suspended in PBS to OD_600_ of 1.0 and diluted 100-fold. One hundred μL of the bacterial suspension was spread on a plate, and sterile disks (Fluka, Germany) soaked with indicated compounds (10 μg EtBr, 750 μg SDS, or 100 μg Vancomycin) were placed on the plates. After three days of incubation, the growth inhibition zone around each disk was measured. Duplicate samples were used and the experiment was repeated twice.

### Stress sensitivity

For stress sensitivity experiments, bacteria were grown in Chamberlain’s medium overnight. For pH stress, bacteria were inoculated into fresh medium adjusted to either pH 4 or 7. For H_2_O_2_ stress, bacteria were subcultured in fresh medium and allowed to grow for another two h before being suspended in PBS containing 0.1 mM of H_2_O_2_, and incubated for 0 or 120 min before dilution series were prepared and plated. For temperature sensitivity, bacteria from overnight cultures were inoculated into fresh medium and incubated until OD_600_ of 1.0 had been reached. The bacterial suspension was then transferred to microcentrifuge tubes and heat shocked at 50°C in a heating block for either 15 or 30 min before dilution series were prepared and plated.

### Transcript analysis

To assess whether all genes from *pdpA* to *pdpE* were part of one transcript, cDNA was prepared from plate grown LVS as described in section “Reverse transcriptase quantitative real-time PCR”. PCR was performed with cDNA as template. Primers used are available upon request.

### Cultivation and infection of macrophages

J774A.1 (J774) mouse macrophage-like cells were used in all cell infection assays, except where otherwise noted. Macrophages were cultured and maintained in DMEM (GIBCO BRL, Grand Island, NY, USA) with 10% heat-inactivated FBS (GIBCO). Peritoneal exudate cells (PEC) were isolated from 8- to 10-week-old C57BL/6 J mice 4 days after intraperitoneal injection of 2 ml of 3% thioglycolate as previously described [21]. Bone marrow derived macrophages (BMDM) were isolated from the femurs and tibias of C57BL/6 J mice essentially as described [17]. For all experiments, cells were seeded in tissue culture plates, incubated overnight, and reconstituted with fresh culture medium at least 30 min prior to infection. A multiplicity of infection (MOI) of 200 was used unless otherwise stated. Plate-grown bacteria were suspended in PBS and kept on ice prior to infection.

### Intracellular immunofluorescence assay

To assess phagosomal escape, GFP-expressing *F*. *tularensis* (using pKK289Km-*gfp*) were used in the cell infections as described previously [18]. Cells were then stained for the LAMP-1 glycoprotein as described previously [12]. Colocalization of GFP-labeled *F*. *tularensis* and LAMP-1 was analyzed with an epifluorescence microscope (ZeissAxioskop2; Carl Zeiss MicroImaging GmbH, Germany). Two biological replicates were used and 100 bacteria per strain and time point were scored. A Student’s *t*-test was used to assess if the colocalization level was significantly different from that of LVS.

### Transmission electron microscopy

Protocol for infection and sample preparation for TEM has been described elsewhere [17]. Sections were viewed with a JEOL JEM 1230 Transmission Electron Microscope (JEOL Ltd., Tokyo, Japan). The membrane integrity was scored by counting at least 100 bacteria from each sample and categorizing each as having: (i) an intact phagosomal membrane, (ii) a slightly damaged phagosomal membrane (< 50% of membrane integrity affected), (iii) a highly damaged phagosomal membrane (> 50% of membrane integrity affected), or (iv) little or no residual membrane (cytoplasmic).

### Intracellular replication in macrophages

Cells were infected with indicated MOI and the infection was allowed to proceed for 2 h followed by washing and addition of fresh cell medium containing 5 μg/ml gentamicin. The number of viable intracellular bacteria at different time points was determined by lysing the monolayers in PBS supplemented with 0.1% deoxycholate and plating serial dilutions on modified GC-agar base plates. A two-sided Student’s *t*-test was used to determine whether the growth of a strain differed significantly from that of LVS.

### RT-qPCR on intracellular bacteria

After infection, J774 murine macrophages were lysed at various time points, by adding one ml Trizol reagent (Ambion, Austin, TX, USA) to each well and scraping with a pipette tip. The suspension was transferred to a 2.0 ml tube and further sample preparation was performed as described earlier in the section “Reverse transcriptase quantitative PCR”. PCR amplification of the 16S gene of *F*. *tularensis* was used as a measure of the number of bacteria, primer sequences have been published elsewhere [31].

### Mouse infections

In order to determine the virulence of *F*. *tularensis* strains, groups of C57BL/6 J female mice (n = 5) were infected intradermally with indicated bacterial doses and mice were examined twice daily for signs of illness, and euthanized by CO_2_ asphyxiation when they showed signs of severe illness, indicating that they were less than 24 h from death. The number of viable bacteria was determined by homogenizing spleens in PBS and plating on GC-agar. All animal experiments were approved by the Local Ethical Committee on Laboratory Animals, Umeå, Sweden (no. A113-08).

### LDH release assay

The LDH release assay has been described in detail elsewhere [17]. In short, cells were infected as described in “Cultivation and infection of macrophages”, at an indicated MOI, washed and new medium added 30 min prior to sampling. Supernatants were collected at indicated time points, and the relative amount of released lactate dehydrogenase was determined using a Cytotox 96 kit (Promega, Madison, WI) according to the manufacturer’s instructions. The results are mean values of six biological replicates and a Student’s *t*-test was used to determine if the difference in LDH release was significant from that of LVS-infected cells. The experiment was repeated three times. Uninfected cells lysed in PBS with 0.1% deoxycholate served as a positive control and was arbitrarily set as 100%; the results were expressed relative to the positive control.

### Data analysis and statistical methods

Statistical significances were determined using paired, two-tailed Student’s *t*-tests.

## Competing interests

The authors declare that they have no competing interests.

## Authors’ contributions

ML, IG and JB generated the constructs and strains used. ML, JB, and LM performed most of the analyses. AS and ML designed the study and drafted the manuscript. All authors read and approved the final manuscript.

## Supplementary Material

Additional file 1: Table S1 Stress sensitivity tests; **Table S2. **Bacterial strains and plasmids; **Table S3. **Primers used in this study. Click here for file

## References

[B1] Bingle LE Bailey CM Pallen MJ Type VI secretion: a beginner’s guide Curr Opin Microbiol 2008 11 1 3 8 10.1016/j.mib.2008.01.006 18289922

[B2] Boyer F Fichant G Berthod J Vandenbrouck Y Attree I Dissecting the bacterial type VI secretion system by a genome wide in silico analysis: what can be learned from available microbial genomic resources? BMC Genomics 2009 10 104 104 19284603 10.1186/1471-2164-10-104 PMC2660368

[B3] Filloux A The type VI secretion system: a tubular story EMBO J 2009 28 4 309 310 10.1038/emboj.2008.301 19225443 PMC2646157

[B4] Hood RD Singh P Hsu F Guvener T Carl MA Trinidad RR Silverman JM Ohlson BB Hicks KG Plemel RL A type VI secretion system of Pseudomonas aeruginosa targets a toxin to bacteria Cell Host Microbe 2010 7 1 25 37 10.1016/j.chom.2009.12.007 20114026 PMC2831478

[B5] Murdoch SL Trunk K English G Fritsch MJ Pourkarimi E Coulthurst SJ The opportunistic pathogen Serratia marcescens utilizes type VI secretion to target bacterial competitors J Bacteriol 2011 193 21 6057 6069 10.1128/JB.05671-11 21890705 PMC3194891

[B6] Russell AB Hood RD Bui NK LeRoux M Vollmer W Mougous JD Type VI secretion delivers bacteriolytic effectors to target cells Nature 2011 475 7356 343 347 10.1038/nature10244 21776080 PMC3146020

[B7] Basler M Pilhofer M Henderson GP Jensen GJ Mekalanos JJ Type VI secretion requires a dynamic contractile phage tail-like structure Nature 2012 483 7388 182 186 10.1038/nature10846 22367545 PMC3527127

[B8] Oyston PC Sjöstedt A Titball RW Tularaemia: bioterrorism defence renews interest in Francisella tularensis Nat Rev Microbiol 2004 2 12 967 978 10.1038/nrmicro1045 15550942

[B9] Bröms JE Sjöstedt A Lavander M The role of the Francisella tularensis pathogenicity island in type VI secretion, intracellular survival, and modulation of host cell signaling Front Microbiol 2010 1 136 136 21687753 10.3389/fmicb.2010.00136 PMC3109350

[B10] Nano FE Schmerk C The Francisella pathogenicity island Ann N Y Acad Sci 2007 1105 122 137 10.1196/annals.1409.000 17395722

[B11] Bröms JE Meyer L Sun K Lavander M Sjöstedt A Unique substrates secreted by the Type VI Secretion System of Francisella tularensis during intramacrophage infection PLoS One 2012 7 11 e50473 10.1371/journal.pone.0050473 23185631 PMC3502320

[B12] Bönquist L Lindgren H Golovliov I Guina T Sjöstedt A MglA and Igl proteins contribute to the modulation of Francisella tularensis live vaccine strain-containing phagosomes in murine macrophages Infect Immun 2008 76 8 3502 3510 10.1128/IAI.00226-08 18474647 PMC2493230

[B13] Chong A Wehrly TD Nair V Fischer ER Barker JR Klose KE Celli J The early phagosomal stage of Francisella tularensis determines optimal phagosomal escape and Francisella pathogenicity island protein expression Infect Immun 2008 76 12 5488 5499 10.1128/IAI.00682-08 18852245 PMC2583578

[B14] de Bruin OM Ludu JS Nano FE The Francisella pathogenicity island protein IglA localizes to the bacterial cytoplasm and is needed for intracellular growth BMC Microbiol 2007 7 1 1 10.1186/1471-2180-7-1 17233889 PMC1794414

[B15] Golovliov I Sjöstedt A Mokrievich A Pavlov V A method for allelic replacement in Francisella tularensis FEMS Microbiol Lett 2003 222 2 273 280 10.1016/S0378-1097(03)00313-6 12770718

[B16] Santic M Molmeret M Klose KE Jones S Kwaik YA The Francisella tularensis pathogenicity island protein IglC and its regulator MglA are essential for modulating phagosome biogenesis and subsequent bacterial escape into the cytoplasm Cell Microbiol 2005 7 7 969 979 10.1111/j.1462-5822.2005.00526.x 15953029

[B17] Bröms JE Lavander M Meyer L Sjöstedt A IglG and IglI of the Francisella pathogenicity island are important virulence determinants of Francisella tularensis LVS Infect Immun 2011 79 9 3683 3696 10.1128/IAI.01344-10 21690239 PMC3165494

[B18] Bröms JE Lavander M Sjöstedt A A conserved a-helix essential for a type VI secretion-like system of Francisella tularensis J Bacteriol 2009 191 8 2431 2446 10.1128/JB.01759-08 19201795 PMC2668417

[B19] Bröms JE Meyer L Lavander M Larsson P Sjöstedt A DotU and VgrG, core components of type VI secretion systems, are essential for Francisella tularensis LVS pathogenicity PLoS One 2012 7 4 e34639 10.1371/journal.pone.0034639 22514651 PMC3326028

[B20] Cole LE Santiago A Barry E Kang TJ Shirey KA Roberts ZJ Elkins KL Cross AS Vogel SN Macrophage proinflammatory response to Francisella tularensis live vaccine strain requires coordination of multiple signaling pathways J Immunol 2008 180 10 6885 6891 18453609 10.4049/jimmunol.180.10.6885 PMC2637793

[B21] Telepnev M Golovliov I Sjöstedt A Francisella tularensis LVS initially activates but subsequently down-regulates intracellular signaling and cytokine secretion in mouse monocytic and human peripheral blood mononuclear cells Microb Pathog 2005 38 5–6 239 247 15925273 10.1016/j.micpath.2005.02.003

[B22] Barker JR Chong A Wehrly TD Yu JJ Rodriguez SA Liu J Celli J Arulanandam BP Klose KE The Francisella tularensis pathogenicity island encodes a secretion system that is required for phagosome escape and virulence Mol Microbiol 2009 74 6 1459 1470 10.1111/j.1365-2958.2009.06947.x 20054881 PMC2814410

[B23] Sjödin A Svensson K Lindgren M Forsman M Larsson P Whole-genome sequencing reveals distinct mutational patterns in closely related laboratory and naturally propagated Francisella tularensis strains PLoS One 2010 5 7 e11556 10.1371/journal.pone.0011556 20657845 PMC2906517

[B24] Twine S Byström M Chen W Forsman M Golovliov I Johansson A Kelly J Lindgren H Svensson K Zingmark C A mutant of Francisella tularensis strain SCHU S4 lacking the ability to express a 58-kilodalton protein is attenuated for virulence and is an effective live vaccine Infect Immun 2005 73 12 8345 8352 10.1128/IAI.73.12.8345-8352.2005 16299332 PMC1307091

[B25] Peng K Broz P Jones J Joubert LM Monack D Elevated AIM2-mediated pyroptosis triggered by hypercytotoxic Francisella mutant strains is attributed to increased intracellular bacteriolysis Cell Microbiol 2011 13 10 1586 1600 10.1111/j.1462-5822.2011.01643.x 21883803 PMC3173570

[B26] Dai S Mohapatra NP Schlesinger LS Gunn JS Regulation of Francisella tularensis virulence Front Microbiol 2010 1 144 21687801 10.3389/fmicb.2010.00144 PMC3109300

[B27] Chong A Celli J The Francisella intracellular life cycle: toward molecular mechanisms of intracellular survival and proliferation Front Microbiol 2010 1 138 138 21687806 10.3389/fmicb.2010.00138 PMC3109316

[B28] Lindgren H Stenmark S Chen W Tarnvik A Sjöstedt A Distinct roles of reactive nitrogen and oxygen species to control infection with the facultative intracellular bacterium Francisella tularensis Infect Immun 2004 72 12 7172 7182 10.1128/IAI.72.12.7172-7182.2004 15557642 PMC529105

[B29] Fortier AH Polsinelli T Green SJ Nacy CA Activation of macrophages for destruction of Francisella tularensis: identification of cytokines, effector cells, and effector molecules Infect Immun 1992 60 3 817 825 1541555 10.1128/iai.60.3.817-825.1992 PMC257560

[B30] Chen W Shen H Webb A KuoLee R Conlan JW Tularemia in BALB/c and C57BL/6 mice vaccinated with Francisella tularensis LVS and challenged intradermally, or by aerosol with virulent isolates of the pathogen: protection varies depending on pathogen virulence, route of exposure, and host genetic background Vaccine 2003 21 25–26 3690 3700 12922099 10.1016/s0264-410x(03)00386-4

[B31] Cole LE Elkins KL Michalek SM Qureshi N Eaton LJ Rallabhandi P Cuesta N Vogel SN Immunologic consequences of Francisella tularensis live vaccine strain infection: role of the innate immune response in infection and immunity J Immunol 2006 176 11 6888 6899 16709849 10.4049/jimmunol.176.11.6888

[B32] Pechous R Celli J Penoske R Hayes SF Frank DW Zahrt TC Construction and characterization of an attenuated purine auxotroph in a Francisella tularensis live vaccine strain Infect Immun 2006 74 8 4452 4461 10.1128/IAI.00666-06 16861631 PMC1539594

[B33] Forslund AL Kuoppa K Svensson K Salomonsson E Johansson A Byström M Oyston PC Michell SL Titball RW Noppa L Direct repeat-mediated deletion of a type IV pilin gene results in major virulence attenuation of Francisella tularensis Mol Microbiol 2006 59 6 1818 1830 10.1111/j.1365-2958.2006.05061.x 16553886

[B34] Lai XH Golovliov I Sjöstedt A Francisella tularensis induces cytopathogenicity and apoptosis in murine macrophages via a mechanism that requires intracellular bacterial multiplication Infect Immun 2001 69 7 4691 4694 10.1128/IAI.69.7.4691-4694.2001 11402018 PMC98551

[B35] Telepnev M Golovliov I Grundström T Tärnvik A Sjöstedt A Francisella tularensis inhibits Toll-like receptor-mediated activation of intracellular signalling and secretion of TNF-alpha and IL-1 from murine macrophages Cell Microbiol 2003 5 1 41 51 10.1046/j.1462-5822.2003.00251.x 12542469

[B36] Gil H Platz GJ Forestal CA Monfett M Bakshi CS Sellati TJ Furie MB Benach JL Thanassi DG Deletion of TolC orthologs in Francisella tularensis identifies roles in multidrug resistance and virulence Proc Natl Acad Sci U S A 2006 103 34 12897 12902 10.1073/pnas.0602582103 16908853 PMC1568944

[B37] Mariathasan S Weiss DS Dixit VM Monack DM Innate immunity against Francisella tularensis is dependent on the ASC/caspase-1 axis J Exp Med 2005 202 8 1043 1049 10.1084/jem.20050977 16230474 PMC2213215

[B38] Jones JW Kayagaki N Broz P Henry T Newton K O’Rourke K Chan S Dong J Qu Y Roose-Girma M Absent in melanoma 2 is required for innate immune recognition of Francisella tularensis Proc Natl Acad Sci U S A 2010 107 21 9771 9776 10.1073/pnas.1003738107 20457908 PMC2906881

[B39] de Bruin OM Duplantis BN Ludu JS Hare RF Nix EB Schmerk CL Robb CS Boraston AB Hueffer K Nano FE The biochemical properties of the Francisella Pathogenicity Island (FPI)-encoded proteins, IglA, IglB, IglC, PdpB and DotU, suggest roles in type VI secretion Microbiology 2011 157 Pt 12 3483 3491 21980115 10.1099/mic.0.052308-0 PMC3352279

[B40] Read A Vogl SJ Hueffer K Gallagher LA Happ GM Francisella genes required for replication in mosquito cells J Med Entomol 2008 45 6 1108 1116 10.1603/0022-2585(2008)45[1108:FGRFRI]2.0.CO;2 19058636

[B41] Åhlund MK Ryden P Sjöstedt A Stöven S A directed screen of Francisella novicida virulence determinants using Drosophila melanogaster Infect Immun 2010 78 7 3118 3128 10.1128/IAI.00146-10 20479082 PMC2897386

[B42] Ulland TK Buchan BW Ketterer MR Fernandes-Alnemri T Meyerholz DK Apicella MA Alnemri ES Jones BD Nauseef WM Sutterwala FS Cutting edge: mutation of Francisella tularensis mviN leads to increased macrophage absent in melanoma 2 inflammasome activation and a loss of virulence J Immunol 2010 185 5 2670 2674 10.4049/jimmunol.1001610 20679532 PMC2953561

[B43] Simeone R Bobard A Lippmann J Bitter W Majlessi L Brosch R Enninga J Phagosomal rupture by Mycobacterium tuberculosis results in toxicity and host cell death PLoS Pathog 2012 8 2 e1002507 10.1371/journal.ppat.1002507 22319448 PMC3271072

[B44] Manzanillo PS Shiloh MU Portnoy DA Cox JS Mycobacterium tuberculosis activates the DNA-dependent cytosolic surveillance pathway within macrophages Cell Host Microbe 2012 11 5 469 480 10.1016/j.chom.2012.03.007 22607800 PMC3662372

[B45] Houben D Demangel C van Ingen J Perez J Baldeon L Abdallah AM Caleechurn L Bottai D van Zon M de Punder K ESX-1-mediated translocation to the cytosol controls virulence of mycobacteria Cell Microbiol 2012 14 8 1287 1298 10.1111/j.1462-5822.2012.01799.x 22524898

[B46] Chamberlain RE Evaluation of live tularemia vaccine prepared in a chemically defined medium Appl Microbiol 1965 13 232 235 14325885 10.1128/am.13.2.232-235.1965 PMC1058227

[B47] Golovliov I Baranov V Krocova Z Kovarova H Sjöstedt A An attenuated strain of the facultative intracellular bacterium Francisella tularensis can escape the phagosome of monocytic cells Infect Immun 2003 71 10 5940 5950 10.1128/IAI.71.10.5940-5950.2003 14500514 PMC201066

[B48] Lindgren H Golovliov I Baranov V Ernst RK Telepnev M Sjöstedt A Factors affecting the escape of Francisella tularensis from the phagolysosome J Med Microbiol 2004 53 Pt 10 953 958 15358816 10.1099/jmm.0.45685-0

[B49] Nano FE Zhang N Cowley SC Klose KE Cheung KK Roberts MJ Ludu JS Letendre GW Meierovics AI Stephens G A Francisella tularensis pathogenicity island required for intramacrophage growth J Bacteriol 2004 186 19 6430 6436 10.1128/JB.186.19.6430-6436.2004 15375123 PMC516616

[B50] Charity JC Costante-Hamm MM Balon EL Boyd DH Rubin EJ Dove SL Twin RNA polymerase-associated proteins control virulence gene expression in Francisella tularensis PLoS Pathog 2007 3 6 e84 10.1371/journal.ppat.0030084 17571921 PMC1891329

[B51] Vallet-Gely I Donovan KE Fang R Joung JK Dove SL Repression of phase-variable cup gene expression by H-NS-like proteins in Pseudomonas aeruginosa Proc Natl Acad Sci U S A 2005 102 31 11082 11087 10.1073/pnas.0502663102 16043713 PMC1182424

[B52] Dove SL Hochschild A A bacterial two-hybrid system based on transcription activation Methods Mol Biol 2004 261 231 246 15064462 10.1385/1-59259-762-9:231

[B53] Kadzhaev K Zingmark C Golovliov I Bolanowski M Shen H Conlan W Sjöstedt A Identification of genes contributing to the virulence of Francisella tularensis SCHU S4 in a mouse intradermal infection model PLoS One 2009 4 5 e5463 10.1371/journal.pone.0005463 19424499 PMC2675058

[B54] Ludu JS de Bruin OM Duplantis BN Schmerk CL Chou AY Elkins KL Nano FE The Francisella pathogenicity island protein PdpD is required for full virulence and associates with homologues of the type VI secretion system J Bacteriol 2008 190 13 4584 4595 10.1128/JB.00198-08 18469101 PMC2446798

