# Supplementary material for: The Francisella tularensis LVS ΔpdpC mutant exhibits a unique phenotype during intracellular infection
Source: BMC Microbiol. 2013 Jan 29;13:20. doi: 10.1186/1471-2180-13-20 (PMC3562505; doi:10.1186/1471-2180-13-20)
Supplement: Additional file 1: Table S1 — Stress sensitivity tests; Table S2. Bacterial strains and plasmids; Table S3. Primers used in this study. [file 1471-2180-13-20-S1.doc]

SUPPLEMENTAL INFORMATION

Supplemental tables

Table S1. Stress sensitivity tests

|  | **pH sensitivity (H_2_O_2_)**^a^ | | | **Temperature sensitivity**^a^ | | | **EtBr**^b^ | **SDS**^b^ | **VA**^b^ |
| --- | --- | --- | --- | --- | --- | --- | --- | --- | --- |
|  | 0 min | 120 min | 240 min | 0 min | 15 min | 30 min | 10 µg | 750 µg | 100 µg |
| LVS | 9.7 | 8.3 | 5.0 | 9.8 | 9.8 | 9.7 | 20 ± 0.0 | 11 ± 0.7 | 10 ± 0.0 |
| ∆*pdpC* | 9.4 | 7.6 | 4.7 | 9.8 | 9.9 | 9.8 | 20 ± 0.7 | 12 ± 0.0 | 10 ± 0.0 |
| ∆*iglA* | 9.7 | 8.1 | 3.6 | 9.7 | 9.8 | 9.7 | N/A | N/A | N/A |

^a^ Results are presented as bacterial growth (log_10_) or ^b^ growth inhibition zone (see Materials and Methods for details).

Table S2. Bacterial strains and plasmids

|  | **Description** | **Reference** |
| --- | --- | --- |
| Strain |  |  |
| *F. tularensis* |  |  |
| LVS | *Francisella tularensis* subsp. *holarctica* Live Vaccine Strain | USAMRIID ^a^ |
| Δ*pdpC* | LVS with in-frame deletion of *pdpC* codons 6-1325 | This study |
| Δ*iglC* | LVS with in-frame deletion of *iglC* codons 28-205 |  |
| Δ*iglA* | LVS with in-frame deletion of *iglA* codons 4-174 |  |
| Δ*pdpC*/*pdpC* | *ΔpdpC* with *pdpC* inserted in *cis* on pDMK3 | This study |
| *E. coli* |  |  |
| Top10 | F- *mcr*A Δ(*mrr-hsd*RMS-*mcr*BC) φ80*lac*ZΔM15 Δ*lac*X74 *rec*A1 *ara*D139 Δ(*araleu*) 7697 *gal*U *gal*K *rps*L (StrR) *end*A1 *nup*G | Invitrogen |
| S17-1λ*pir* | *recA*, *thi*, *pro*, *hsdR^-^M^+^*, Sm^R^, <RP4:2-Tc:Mu:Ku:Tn7>Tp^R^ |  |
|  |  |  |
| DH5αF′IQ | F-φ80*lac*ZΔM15 Δ(*lac*ZYA-*arg*F) U169 *rec*A1 *end*A1 *hsd*R17 (rk-, mk+) *pho*A *sup*E44 λ- *thi*-1 *gyr*A96 *rel*A1/F´ *pro*AB+ *lac*IqZΔM15 zzf::Tn5 [Km^R^] | Invitrogen |
| KDZif1ΔZ | B2H reporter strain, Km^R^, Cml^R^ |  |
| Plasmid |  |  |
| pCR®4-TOPO® | Topo cloning vector, Amp^R^, Km^R^ | Invitrogen |
| pDMK3 | pDM4 derivative, Km^R^ |  |
| pKK289Km | pKK214 derivative encoding *gfp*, Km^R^ |  |
| pACTR-AP-Zif | B2H vector, directs the synthesis of a Zif268-DNA binding domain fusion protein, Tet^R^ |  |
| pJEB871 | pACTR-AP-Zif encoding DotU, Tet^R^ |  |
| pJEB864 | pACTR-AP-Zif encoding FevR, Tet^R^ | This study |
| pJEB876 | pACTR-AP-Zif encoding IcmF, Tet^R^ |  |
| pMOL135 | pACTR-AP-Zif encoding IglA, Tet^R^ |  |
| pMOL139 | pACTR-AP-Zif encoding IglB, Tet^R^ |  |
| pLM5 | pACTR-AP-Zif encoding IglC, Tet^R^ |  |
| pACTR-IglD | pACTR-AP-Zif encoding IglD, Tet^R^ | This study |
| pACTR-IglE | pACTR-AP-Zif encoding IglE, Tet^R^ | This study |
| pJEB828 | pACTR-AP-Zif encoding IglF, Tet^R^ | This study |
| pACTR-IglG | pACTR-AP-Zif encoding IglG, Tet^R^ | This study |
| pACTR-IglH | pACTR-AP-Zif encoding IglH, Tet^R^ | This study |
| pACTR-IglI | pACTR-AP-Zif encoding IglI, Tet^R^ | This study |
| pACTR-IglJ | pACTR-AP-Zif encoding IglJ, Tet^R^ | This study |
| pACTR-MglA-Zif | pACTR-AP-Zif encoding MglA, Tet^R^ |  |
| pSK011 | pACTR-AP-Zif encoding PdpA, Tet^R^ | This study |
| pACTR-PdpC | pACTR-AP-Zif encoding PdpC, Tet^R^ | This study |
| pSK012 | pACTR-AP-Zif encoding PdpD, Tet^R^ | This study |
| pACTR-PdpE | pACTR-AP-Zif encoding PdpE, Tet^R^ | This study |
| pJEB862 | pACTR-AP-Zif encoding PmrA, Tet^R^ | This study |
| pJEB873 | pACTR-AP-Zif encoding VgrG, Tet^R^ |  |
| pBRGPω | B2H vector, directs the synthesis of a Gal11P-ω fusion protein, Cb^R^ |  |
| pJEB872 | pBRGPω encoding DotU, Cb^R^ |  |
| pJEB865 | pBRGPω encoding FevR, Cb^R^ | This study |
| pJEB877 | pBRGPω encoding IcmF, Cb^R^ |  |
| pMOL133 | pBRGPω encoding IglA, Cb^R^ |  |
| pMOL134 | pBRGPω encoding IglB, Cb^R^ |  |
| pLM6 | pBRGPω encoding IglC, Cb^R^ |  |
| pBRG-IglD | pBRGPω encoding IglD, Cb^R^ | This study |
| pBRG-IglE | pBRGPω encoding IglE, Cb^R^ | This study |
| pJEB829 | pBRGPω encoding IglF, Cb^R^ | This study |
| pBRG-IglG | pBRGPω encoding IglG, Cb^R^ | This study |
| pBRG-IglH | pBRGPω encoding IglH, Cb^R^ | This study |
| pBRG-IglI | pBRGPω encoding IglI, Cb^R^ | This study |
| pBRG-IglJ | pBRGPω encoding IglJ, Cb^R^ | This study |
| pSK013 | pBRGPω encoding PdpA, Cb^R^ | This study |
| pBRG-PdpC | pBRGPω encoding PdpC, Cb^R^ | This study |
| pSK014 | pBRGPω encoding PdpD, Cb^R^ | This study |
| pBRG-PdpE | pBRGPω encoding PdpE, Cb^R^ | This study |
| pJEB863 | pBRGPω encoding PmrA, Cb^R^ | This study |
| pBRSspA-ω | pBRGPω encoding SspA, Cb^R^ |  |
| pJEB874 | pBRGPω encoding VgrG, Cb^R^ |  |

^a^ Obtained from The United States Army Medical Research Institute for Infectious Diseases, 1425 Porter Street, Frederick, MD, USA.

TABLE S3. Primers used in this study

| Purpose | Primer pairs |
| --- | --- |
| *LVS null mutant* |  |
| PdpC Δ6-1325 | PdpC_OF: 5´-ATACAAGGCTCTGAGAAATGGAAAACTCTT-3´ and PdpC_IR: 5´-AACCTATGATG*AATATT*TGTCGTTCATATGTACCTCCTTAAT-3´  PdpC_IF: 5´-AACGACA*AATATT*CATCATAGGTTAAGGATACAAATATATGA-3´ and PdpC_OR: 5´-GTTAGATAGAGGGCGATTAGTACCAGAAAT-3´ |
| *Complementation* |  |
| PdpC (cis) | pdpC_SalI_cis-F: 5´-GGT*GTCGAC*AAGGCTCTGAGAAATGGAAA-3´ and pdpC_SpeI_cis-R: 5´-GG*ACTAGT*CGAATGTACTAGCTGTTATTGTA-3´ |
| *B2H constructs* |  |
| FevR | FTL0499_NdeI_F: 5´-*CATATG*GCGAATCAATATTCTGGAA-3´and FTL0499_b: 5´-**A**GCTATCTCTCTTTTTTGTTCAAGA-3´ FTL0499_c: 5´-AAAAAGAGAGATAGC**T**TATGTCAATCAATGTAAGTTGAATAA-3´ and FTL0499_NotI_R: 5´-*GCGGCCGC*AGATTTAGCTTTGATTACAGAATA-3´ |
| IglD | IglD_Y2H_F: 5´-*CATATG*TTTCTAGAAAGGATTTATTGGGAAGAT-3´ and IglD_NotI_rev: 5´-*GCGGCCGC*AGAAAAGGCTATAAAGAAATCAA-3´ |
| IglE | IglE_F_NdeI2: 5´-*CATATG*TACAATAAATTATTGAAAAATCTTtgtttagtA-3´and IglE_NotI_rev: 5´-*GCGGCCGC*ATCTTTTTCTATGCTACTATCA-3´ |
| IglF | IglF_NdeI_for: 5´-*CATATG*AATAATAATATTGATAAATGGTTTGA-3´ and IglF_NotI_R: 5´-*GCGGCCGC*AGCACCAAAAAAAAAACTATTTGAAATTG-3´ |
| IglG | pigDfor: 5´-*CATATG*TTAAATATTATAAATGACTCC-3´and IglG_NotI_rev: 5´-*GCGGCCGC*AGATGTTTTTACATTTATTTGTCCA-3´ |
| IglH | FTL0120_NdeI_F: 5´-*CATATG*GATGAAAAAAGAAAAGATTTAAG-3´and FTL0120_NotI_R: 5´-*GCGGCCGC*TATAGAGTTATTTAAAACAATCTTTTTAA-3´ |
| IglI | pigG_for: 5´-*CATATG*AGTCAGATAATATCTACAC-3´ and IglI_NotI_rev: 5´-*GCGGCCGC*TATGTCAAAAAGATCTTCAAAATAG-3´ |
| IglJ | IglJ_NdeI_fw: 5´-*CATATG*AAGACTATTTTGAAGATCTT-3´ and IglJ_mut_rev: 5´-**A**GAAGGAATATATGCCCCCAA-3´ IglJ_mut_F: 5´-GCATATATTCCTTC**T**TATGTTTATATTATAAACATTAAG-3´ and IglJ_NotI_rev: 5´-*GCGGCCGC*TAAATTAAAATAACTTAGGTATATC-3´ |
| PdpA | Y2H_pdpA1F: 5´-*CAT ATG* ATA GCA GTA AAA GAT ATA ACT GAT-3´ and PdpA_NotI_R: 5´-*GCG GCC GC*A TTT CCT TTT GAT TTA TAT CTT AG-3´ |
| PdpC | FTL_0116_NdeI: 5´-*CATATG*AACGACAAATATGAACTAAAT-3´and FTL_0116_NotI: 5´-*GCGGCCGC*TGATGATATTTTTTTAAAAAAGTCTGAT-3´ |
| PdpD | PdpD_NdeI_F: 5´-*CAT ATG* GAT CAA GAT ATC AAC GAT TTA TTA T-3´ and PdpD_NdeImut_R: 5´-AAA CAT **G**TG TCT TTC AAC GTC AT-3´  PdpD_NdeImut_F: 5´-TGA CGT TGA AAG ACA **C**AT GTT T-3´ and PdpD_NotI_R: 5´- *GCG GCC GC* AAC CCA GAT CAT TGG TCT ATA CTT T -3´ |
| PdpE | pigI_for:5´-*CATATG*AGTAAAAAAATATTTAAATTATTATCAAT-3´ and PdpE_NotI_rev:5´-*GCGGCCGC*TATTATAGTAATTTTCTTTTCATAATGA-3´ |
| PmrA | FTL0552_NdeI_F: 5´-*CATATG*AGAATATTGTTGGCTGAAGA-3´and FTL0552_b: 5´-**G**TGTACTTCTAGGGTGTTTGTATC-3´  FTL0552_c: 5´-CACCCTAGAAGTACA**C**ATGCATAATTTAAGAAAGAAAATTA-3´ and FTL0552_NotI_R: 5´-*GCGGCCGC*CTTAATTACTTTATCCTTTTGTACA-3´ |

The nucleotide sequences in italics represent the incorporated restriction sites (*SspI*, *SalI*, *SpeI, Nde*I, *Not*I) used for cloning of the PCR amplified DNA fragments. The underlined sequence indicates the complementary overlap between respective primers in the overlap PCR reactions. In primers used to generate amino acid substitutions, the nucleotides substituted are indicated in boldface. To optimize expression, all substitutions were adapted according to the codon usage preferences of *F*. *tularensis* (http://www.kazusa.or.jp/codon)

1. Golovliov I, Sjöstedt A, Mokrievich A, Pavlov V: **A method for allelic replacement in *Francisella tularensis***. *FEMS Microbiol Lett* 2003, **222**(2):273-280.

2. Bröms JE, Lavander M, Sjöstedt A: **A conserved a-helix essential for a type VI secretion-like system of *Francisella tularensis***. *J Bacteriol* 2009, **191**(8):2431-2446.

3. Simon R, Priefer U, Pühler A: **A broad host range mobilisation system for *in vivo* genetic engineering: transposon mutagenesis in Gram negative bacteria**. *Biotechnology* 1983, **1**:787-796.

4. Vallet-Gely I, Donovan KE, Fang R, Joung JK, Dove SL: **Repression of phase-variable cup gene expression by H-NS-like proteins in *Pseudomonas aeruginosa***. *Proc Natl Acad Sci U S A* 2005, **102**(31):11082-11087.

5. Lindgren H, Shen H, Zingmark C, Golovliov I, Conlan W, Sjostedt A: **Resistance of *Francisella tularensis* Strains against Reactive Nitrogen and Oxygen Species with Special Reference to the Role of KatG**. *Infect Immun* 2007, **75**(3):1303-1309.

6. Bönquist L, Lindgren H, Golovliov I, Guina T, Sjöstedt A: **MglA and Igl proteins contribute to the modulation of *Francisella tularensis* live vaccine strain-containing phagosomes in murine macrophages**. *Infect Immun* 2008, **76**(8):3502-3510.

7. Charity JC, Costante-Hamm MM, Balon EL, Boyd DH, Rubin EJ, Dove SL: **Twin RNA polymerase-associated proteins control virulence gene expression in *Francisella tularensis***. *PLoS Pathog* 2007, **3**(6):e84.

8. Bröms JE, Meyer L, Lavander M, Larsson P, Sjöstedt A: **DotU and VgrG, core components of type VI secretion systems, are essential for *Francisella tularensis* LVS pathogenicity**. *PloS one* 2012, **7**(4):e34639.
